# DNA hypomethylation promotes the expression of CASPASE-4 which exacerbates inflammation and amyloid-β deposition in Alzheimer’s disease

**DOI:** 10.1186/s13195-024-01390-2

**Published:** 2024-02-08

**Authors:** Kylene P. Daily, Asmaa Badr, Mostafa Eltobgy, Shady Estfanous, Owen Whitham, Michelle H. Tan, Cierra Carafice, Kathrin Krause, Andrew McNamara, Kaitlin Hamilton, Samuel Houle, Spandan Gupta, Gauruv A. Gupta, Shruthi Madhu, Julie Fitzgerald, Abbey A. Saadey, Brooke Laster, Pearlly Yan, Amy Webb, Xiaoli Zhang, Maciej Pietrzak, Olga N. Kokiko-Cochran, Hazem E. Ghoneim, Amal O. Amer

**Affiliations:** 1 Department of Microbial Infection and Immunity, Infectious Diseases Institute, The Heart and Lung Research Institute, The Ohio State University, Columbus, OH 43210 USA; 2 Clinical Pathology Department, College of Medicine, Mansoura University, Mansoura, Egypt; 3 Biochemistry and Molecular Biology Department, Faculty of Pharmacy, Helwan University, Cairo, Egypt; 4 Max Planck Unit for the Science of Pathogens, Berlin, Germany; 5 Department of Neuroscience, The Ohio State University, Columbus, OH 43210 USA; 6 Genomics Shared Resource, Department of Internal Medicine, Comprehensive Cancer Center, The Ohio State University, Columbus, OH 43210 USA; 7 Department of Biomedical Informatics, The Ohio State University, Columbus, OH USA; 8 Center for Biostatistics, Ohio State University, Columbus, OH USA; 9 Pelotonia Institute for Immuno-Oncology, James Comprehensive Cancer Center, The Ohio State University, Columbus, OH USA

**Keywords:** Alzheimer’s disease, Inflammasome, Inflammation, Caspase-4, Caspase-11, Methylation, Amyloid-β, IL-1β, Neuroinflammation

## Abstract

Alzheimer’s disease (AD) is the sixth leading cause of death in the USA. It is established that neuroinflammation contributes to the synaptic loss, neuronal death, and symptomatic decline of AD patients. Accumulating evidence suggests a critical role for microglia, innate immune phagocytes of the brain. For instance, microglia release pro-inflammatory products such as IL-1β which is highly implicated in AD pathobiology. The mechanisms underlying the transition of microglia to proinflammatory promoters of AD remain largely unknown. To address this gap, we performed reduced representation bisulfite sequencing (RRBS) to profile global DNA methylation changes in human AD brains compared to no disease controls. We identified differential DNA methylation of CASPASE-4 (CASP4), which when expressed promotes the generation of IL-1β and is predominantly expressed in immune cells. DNA upstream of the CASP4 transcription start site was hypomethylated in human AD brains, which was correlated with increased expression of CASP4. Furthermore, microglia from a mouse model of AD (5xFAD) express increased levels of CASP4 compared to wild-type (WT) mice. To study the role of CASP4 in AD, we developed a novel mouse model of AD lacking the mouse ortholog of CASP4 and CASP11, which is encoded by mouse *Caspase-4* (5xFAD/*Casp4*^*−/−*^). The expression of CASP11 was associated with increased accumulation of pathologic protein aggregate amyloid-β (Aβ) and increased microglial production of IL-1β in 5xFAD mice. Utilizing RNA-sequencing, we determined that CASP11 promotes unique transcriptomic phenotypes in 5xFAD mouse brains, including alterations of neuroinflammatory and chemokine signaling pathways. Notably, in vitro, CASP11 promoted generation of IL-1β from macrophages in response to cytosolic Aβ through cleavage of downstream effector Gasdermin D (GSDMD). Therefore, here we unravel the role for CASP11 and GSDMD in the generation of IL-1β in response to Aβ and the progression of pathologic inflammation in AD. Overall, our results demonstrate that overexpression of CASP4 due to differential DNA methylation in AD microglia contributes to the progression of AD pathobiology. Thus, we identify CASP4 as a potential target for immunotherapies for the treatment and prevention of AD.

**Supplementary Information:**

The online version contains supplementary material available at 10.1186/s13195-024-01390-2.

## Introduction

Alzheimer’s disease (AD) is a progressive neurodegenerative disorder and the sixth leading cause of death in the USA [1]. Currently, there are no reliable preventative methods and very limited potential treatment options for AD [2]. Brain pathology in AD is characterized by extracellular senile plaques of amyloid-beta (Aβ) and intracellular neurofibrillary tangles of tau protein. An additional hallmark of AD is neuroinflammation, which contributes to the synaptic loss, neuronal death, and symptomatic decline of AD patients [3]. Neuroinflammation in AD is coordinated by progressive changes in brain inflammatory cells, such as microglia and brain-associated macrophages [4]. The changes in microglia responses are regulated via epigenetic mechanisms [5, 6]. In particular, DNA methylation—an epigenetic mechanism that controls stable gene expression programs—is globally deregulated in neurodegenerative diseases such as AD [7, 8]. Therefore, it is plausible that the progressive inflammatory response seen in AD coincides with altered methylation status of critical immune effector molecules.

Amyloid beta (Aβ) is known to promote the production of inflammatory cytokines including IL-1β from microglia and macrophages [9], contributing to chronic long-lasting sterile neuroinflammation in AD. For instance, the potent inflammatory cytokine IL-1β is implicated in AD pathogenesis by a variety of mechanisms. Multiple IL-1β genetic polymorphisms are associated with AD [10, 11]. IL-1β stimulates increased Aβ production from neurons and exacerbates neurofibrillary tangle formation [12–15]. Thus, identifying the mediators involved in neuroinflammation and the production of IL-1β will provide mechanistic insight and potential diagnostic and therapeutic targets for AD.

Inflammatory mouse Caspase-11 (CASP11) and human orthologues Caspase-4 (CASP4) and Caspase-5 (CASP5) are the main drivers of noncanonical inflammasome activation which promotes release of IL-1β [16]. We and others demonstrated that human CASP4 performs most of the functions of mouse CASP11, and for simplicity, we will refer to them as CASP4 for human and CASP11 for mouse [17, 18]. As both mouse and human proteins are encoded by the *Caspase-4 gene*, we refer to the gene for both as *Casp4*. In various biologic contexts, CASP4 promotes cleavage of Caspase-1 (CASP1) which activates IL-1β [16, 19–21]. Once cleaved, GSDMD forms pores in the plasma membrane, which allows the release of active IL-1β. The extensive formation of GSDMD pores can trigger inflammatory cell death and pyroptosis [22, 23]. GSDMD can be cleaved by active CASP1 or CASP11 according to the insult [20, 21]. Yet, it is currently unclear if CASP11 and GSDMD are required for CASP1 activation and inflammatory responses to Aβ. Importantly, CASP4 is upregulated in the brain of AD patients and correlates with disease progression and expression of risk genes [24, 25]. However, it remains largely unknown whether the increased expression of CASP4 is influenced by epigenetic factors or is associated with occurrence of hallmarks of AD pathobiology.

In this study, we profiled the global DNA methylation programs in brain tissues from AD patients and no disease controls. Importantly, we identified a unique DNA demethylation program upstream of the *CASP4* transcription start site in AD patients, which is correlated with an increased expression of CASP4 in human AD. To further understand the role of CASP4 in the development of AD, we developed a mouse model of AD (5xFAD, five familial Alzheimer’s disease mutations) lacking CASP11. We found that CASP11 drives Aβ deposition in male mice. Additionally, expression of CASP11 promoted release of IL-1β from microglia of 5xFAD mouse brains. Furthermore, transcriptomic profiling of the hippocampal tissue from 5xFAD/*Casp4*^*−/−*^ mice revealed that CASP4 promotes neuroinflammation and chemokine signaling in 5xFAD mice. Finally, we performed in vitro analysis of inflammasome activation in response to Aβ introduced to cells by a cytosolic-delivery reagent in primary macrophages. We found that fibrillar-Aβ activates the inflammasome response and that both CASP11 and NLR family pyrin domain-containing 3 (NLRP3) drive IL-1β activation and release. Our work positions CASP4 as a novel regulator of microglia inflammation in AD and demonstrates a molecular mechanism underlying its increased expression in AD brains.

## Results

### CASP4 upregulation is coupled with DNA demethylation events in the brains of human subjects with Alzheimer’s disease

**Fig. 1:**
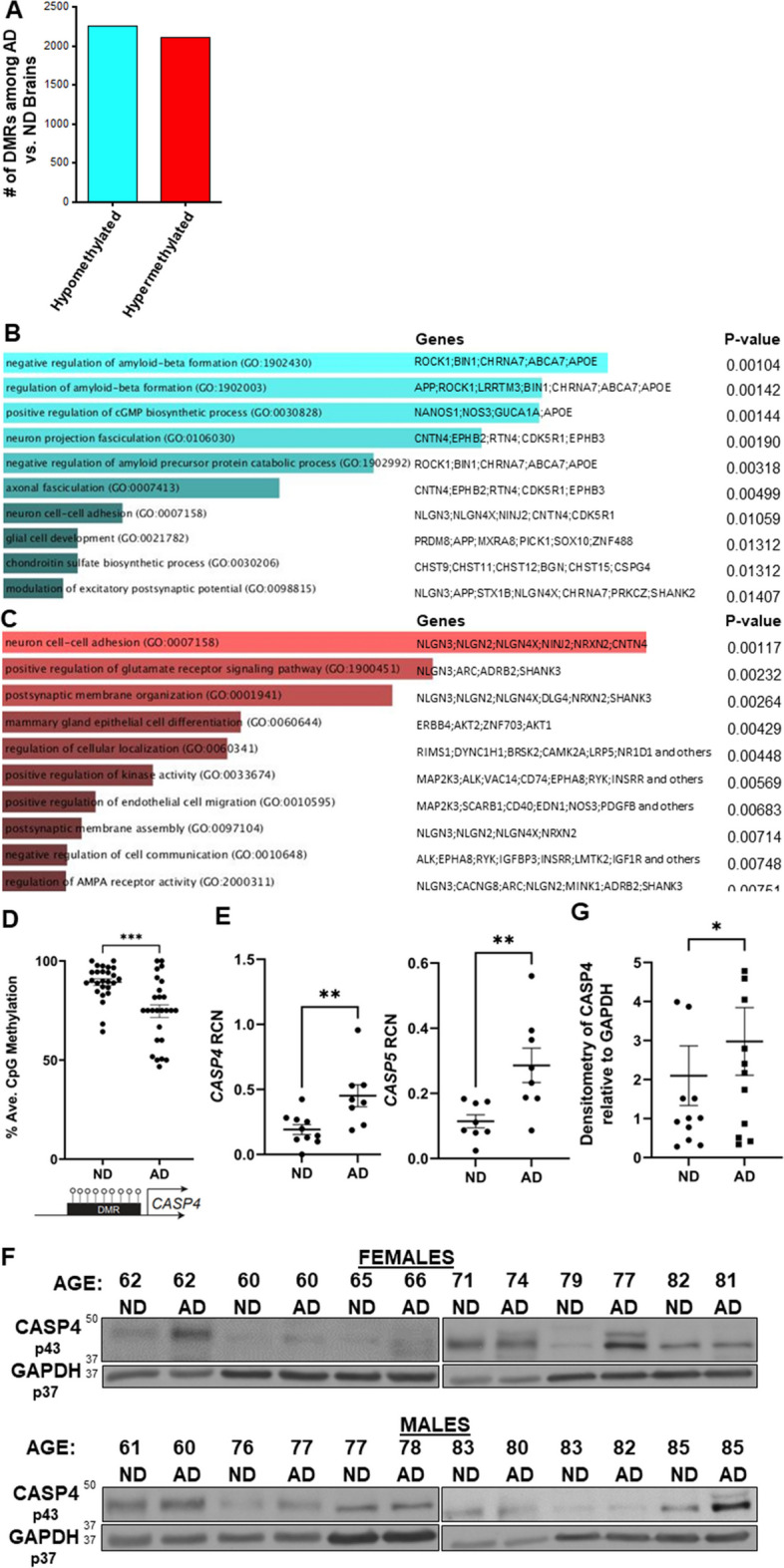
Human Alzheimer’s disease brains undergo distinct changes in DNA methylation including at the CASP4 locus. **A** Numbers of differentially methylated regions (DMRs) identified by RRBS when comparing Alzheimer’s disease (AD) brains to non-dementia (ND) brains. **B**–**C** Top GO biologic processes and enriched genes with hypomethylated (**B**) or hypermethylated (**C**) DMRs. **D** Average CpG methylation in temporal lobes of human Alzheimer’s disease (AD) and non-dementia (ND) controls for CASP4 differentially methylated region (DMR). Selected DMR is located ~ 50 base pair upstream of the CASP4 transcription start site. Unpaired *t*-test with Welch’s correction (*N* = 3 individual brain samples). *P ≤ 0.05, **P ≤ 0.01, ***P ≤ 0.001. **E** Relative copy number (RCN) of CASP4 and CASP5 in temporal lobes of human AD and non-dementia controls determined by RT-PCR. Unpaired *t*-test (*N* = 8). **F** Immunoblot of CASP4 in temporal lobes of human AD and no disease (ND) controls. **G** Densitometry of CASP4 relative to GAPDH above background for immunoblots in **F**. Unpaired *t*-test (*N* = 12)

To better understand the DNA methylation deregulation during AD, we performed reduced representation bisulfite sequencing (RRBS) to profile global DNA methylation changes within frozen brain tissues at the temporal lobe (Brodmann area 38) from human AD brains and age- and sex-matched no disease (ND) controls. The temporal lobe is often the location where the characteristic spread of AD pathology including Aβ plaques starts [26]. We found > 4000 differentially methylated regions (DMRs) that were either hypomethylated or hypermethylated in AD versus ND brains (Fig. 1A; Supplementary Data 1). This data is shared through Gene Expression Omnibus with accession number GSE227194. To gain insights into the functional significance of these DMRs, we performed Gene Ontology (GO) enrichment analysis of the differentially methylated genes and found that hypomethylated DMRs in human brains with AD are enriched in biological processes regulating AD pathogenesis, such as Aβ formation (Fig. 1B). In contrast, the hypermethylated DMRs in AD brains are enriched in biological processes regulating glutamate receptor signaling and neuronal cell–cell adhesion (Fig. 1C). These findings indicate a significant role of DNA methylation programming in regulating the AD progression in human brains. When we examined DNA methylation changes within *CASP4* loci, we identified a unique hypomethylated DMR located ~ 350-bp upstream of the transcription start site at the CASP4 locus in AD patients as compared to patients without disease (*P*-value = 1.47, E-6; *n* = 5). To further validate the differential methylation state in AD brain samples, we designed a targeted epigenetic assay to assess DNA methylation levels at individual CpG sites in that genomic region [27]. We found significant reduction in the average CpG methylation levels within this DMR in AD patients compared to non-dementia patients (Fig. 1D). This DNA demethylation program was coupled with increased transcript and protein expression levels of CASP4 in human AD samples compared to non-dementia controls (Fig. 1E–G). Notably, we found no difference in expression based on sex, when comparing the expression levels of CASP4 protein among human male and female brain samples (Fig. 1F). These data indicate that CASP4 is upregulated in human brains with AD, which is epigenetically regulated, at least in part, by DNA demethylation programming.

### CASP11 is upregulated in 5xFAD mice specifically in microglia

**Fig. 2:**
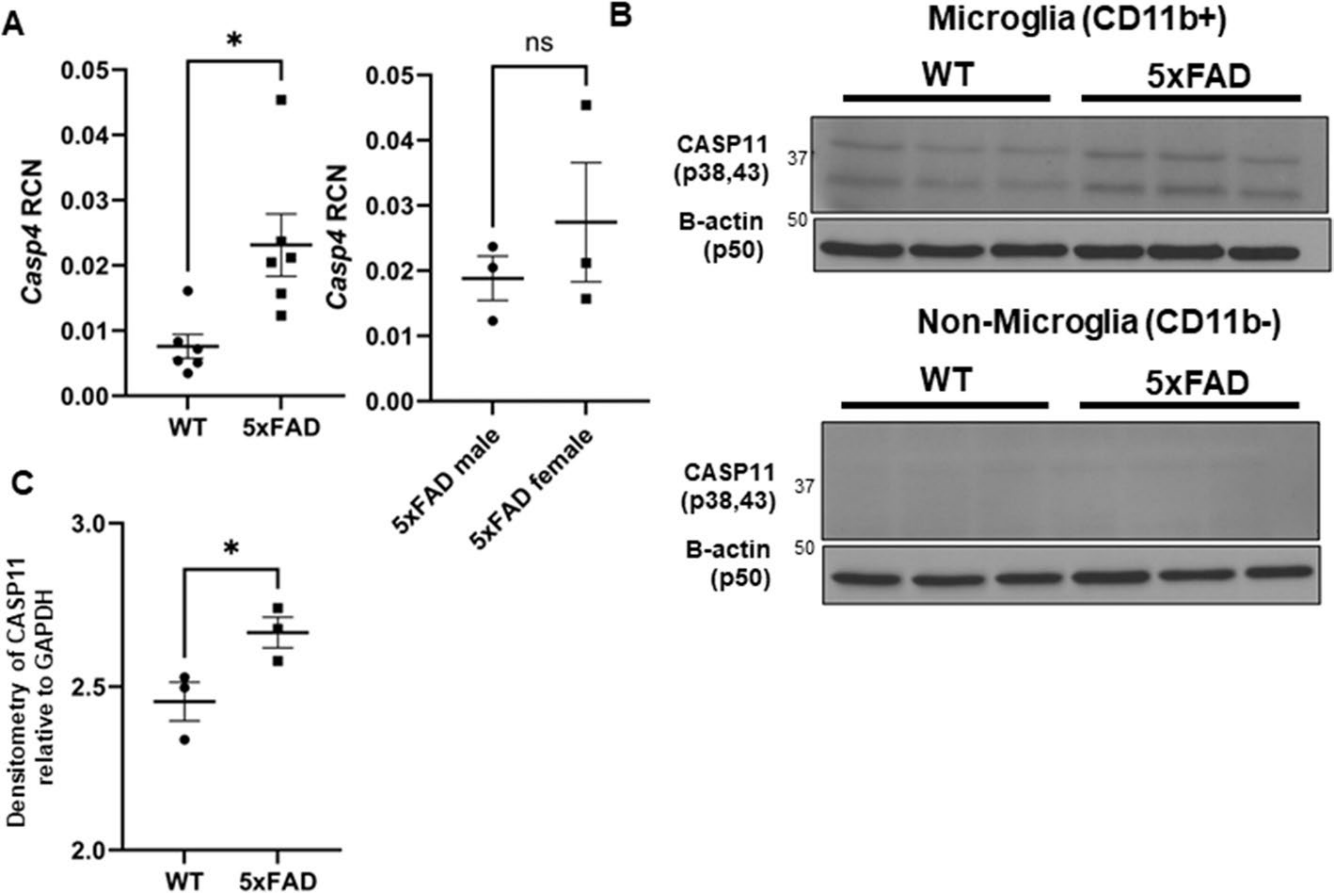
CASP11 exhibits increased expression in the 5xFAD microglia. **A** Relative copy number (RCN) of *Casp4* in homogenized mouse brains from 7-month-old 5xFAD and age- and sex-matched wild-type (WT) littermate controls and 5xFAD male mice compared to female mice determined by RT-PCR. RCNs are relative to housekeeping gene *GAPDH* and multiplied by a factor of 100. Statistical analysis was completed by Students *t*-test. **B** Immunoblots for CASP11 and β-actin loading control in microglia (CD11b +) and non-microglia (CD11b −) fractions from 5-month-old WT and 5xFAD mice. **C** Densitometry of CASP11 relative to GAPDH above background in microglia (CD11b +) for immunoblot shown in **B** (*N* = 3). Statistical analysis completed by Student’s *t*-test. *P ≤ 0.05

We then determined if CASP11 expression was increased in the 5xFAD mouse model. The 5xFAD mouse model expresses five mutations detected in familial forms of AD under the Thy1 neuronal promoter [28]. This model exhibits robust deposition of Aβ, starting at 2 months of age, followed by microgliosis and loss of cognitive function around 6 months [28]. Expression of mouse *Casp4* which encodes CASP11 in the hippocampus of 7-month-old 5xFAD mice was analyzed by quantitative real-time PCR (RT-qPCR). 5xFAD mice express significantly more *Casp4* than age- and sex-matched wild-type (WT) littermates (Fig. 2A). Importantly, there was no significant difference in the level of *Casp4* expression in 5xFAD male mice compared to female mice (Fig. 2A). In other organs, *Casp4* is predominantly expressed in macrophages in response to DAMPs or PAMPs [29, 30]. Since microglia are considered the main phagocytic immune cells of the brain, the expression of CASP11 in both 5xFAD microglia and non-microglia fractions was analyzed. Microglia were isolated from homogenized whole mouse brains by magnetic bead-positive selection for CD11b to analyze expression of CASP11 by immunoblot. The non-microglia (CD11b −) fraction was also collected and contains all cell types other than microglia, such as neurons, endothelial cells, astrocytes, oligodendrocytes, and ependymal cells as described previously [31]. Microglia from 5xFAD mice expressed higher levels of CASP11 than age- and sex-matched WT mice (Fig. 2B–C). There was no measurable expression of CASP11 in the combined non-microglia cells (Fig. 2B). To further understand the cellular expression pattern of *Casp4* in the brain, we utilized a recently published pool of scRNA-seq datasets of immune cells in the mouse brain under homeostasis and analyzed the expression of *Casp4* expression levels in this scRNA-seq atlas [32]. In agreement with our findings, we found that *Casp4* is mainly/highly expressed by microglia which also highly express CX3CR1 (Supplementary Fig. 1). Therefore, we concluded that the expression of CASP11 is increased specifically in microglia of 5xFAD mice.

### The expression of CASP11 drives brain pathology and inflammation in 5xFAD mouse brains

**Fig. 3:**
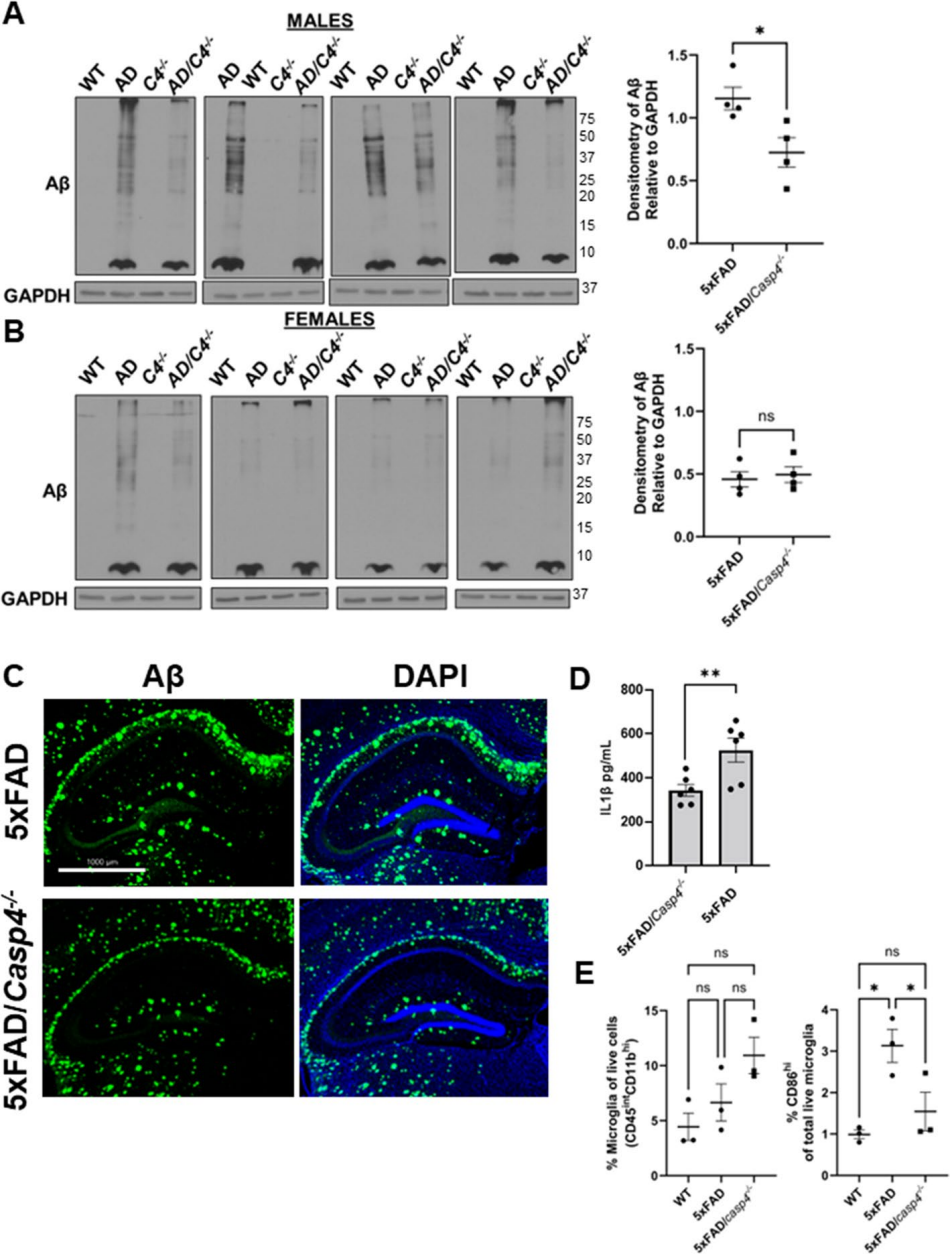
CASP11 deficiency leads to decreased Aβ deposition and reduced microglial release of IL-1β in 5xFAD mice. Immunoblots for Aβ and GAPDH loading control in hippocampal lysate from 7-month-old littermate WT, 5xFAD, *Casp4*^*−/−*^, and 5xFAD/*Casp4*^*−/−*^ for **A** male and **B** female mice. Densitometry analysis of immunoblots for Aβ relative to GAPDH above background is displayed. Statistical analysis completed by Student’s *t*-test. *P ≤ 0.05. **C** Immunohistochemistry of Aβ (green) and DAPI (blue) in the hippocampus of 5xFAD and 5xFAD/*Casp4*^*−/−*^ mice. **D** IL-1β in cell culture supernatants from 5 and 5xFAD/*Casp4*^*−/−*^ microglia from 8- to 10-month-old male and female mice. Statistical analysis completed mixed-effect analysis with Sidak multiple comparison test (*N* = 6). *P < 0.05, **P < 0.005. **E** Percentage of microglia (CD45^int^ CD11b^high^) among the live cell population and percentage of microglia expressing high levels of CD86 from whole brain homogenates 7-month-old littermate WT, 5xFAD, and 5xFAD/*Casp4*.^*−/−*^ mice. Statistical analysis for completed by two-way ANOVA Tukey’s multiple comparisons test. *P ≤ 0.05

Since disease progression in the 5xFAD mouse correlates with increasing deposition of Aβ plaques [33], we characterized Aβ burden in the 5xFAD mice lacking the *Casp4* gene (which encodes mouse CASP11 protein). To evaluate the role of CASP11 in 5xFAD brain pathophysiology, we crossed *Casp4*^*−/−*^ mice with 5xFAD mice. We also maintained generation-matched WT/*Casp4*^+*/*+^ (WT), WT/*Casp4*^*−/−*^ (*Casp4*^*−/−*^), and 5xFAD/*Casp4*^+*/*+^ (5xFAD) littermate mice. We quantified Aβ within the entire hippocampus of both 7-month-old male and female mice. To do so, the hippocampus was dissected, snap frozen, and cryopulverized. We found Aβ is significantly reduced in male 5xFAD/*Casp4*^*−/−*^ mice compared to 5xFAD (Fig. 3A). However, in female mice we did not see a consistent reduction in Aβ by immunoblot (Fig. 3B). As expected, we did not detect Aβ within the WT or *Casp4*^*−/−*^ mice. Immunohistochemical staining of Aβ is displayed to demonstrate distribution of Aβ within the hippocampus of 5xFAD/*Casp4*^*−/−*^ mice compared to 5xFAD mice (Fig. 3C).

It is well-described that AD is an inflammatory disease, and that potent inflammatory cytokine IL-1β stimulates increased Aβ production [12–15]. Therefore, we determined if IL-1β release from microglia was exacerbated by the expression of CASP11. To do this, we isolated and cultured microglia from 8- to 10-month-old 5xFAD and 5xFAD/*Casp4*^*−/−*^ mice. Microglia were incubated for 24 h after treatment with inflammatory stimulus lipopolysaccharide (LPS). We found that microglia from both male and female mice released more IL-1β when CASP11 was expressed (Fig. 3D). To evaluate the role of CASP11 in regulation of activation status of microglia, we analyzed microglia for expression of pro-inflammatory microglia activation marker CD86 [34]. To do this, we utilized flow cytometric analysis of the microglia population (CD45^int^CD11b^high^) of whole WT, 5xFAD, and 5xFAD/*Casp4*^*−/−*^ brains. There was no difference in the percentage of microglia among the live cell population (Fig. 3E). We found that more microglia from 5xFAD brains expressed pro-inflammatory marker CD86 than microglia from WT and 5xFAD brains lacking CASP11 (Fig. 3E). Taken together, we concluded that the expression of CASP11 drives Aβ deposition in male 5xFAD mice and microglial activation and release of IL-1β.

### CASP11 promotes neuroinflammation and chemokine production in 5xFAD mice

**Fig. 4:**
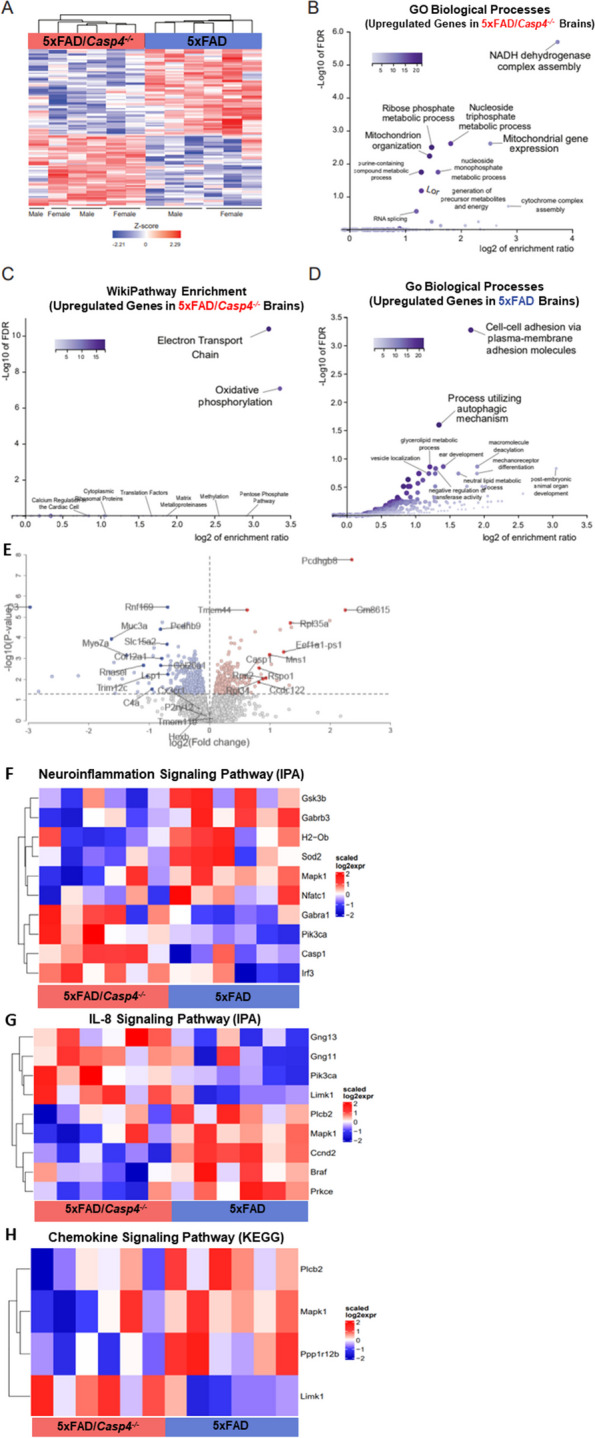
CASP11 promotes unique genetic phenotype including alterations of neuroinflammatory signaling pathways in the 5xFAD hippocampus. **A** Heatmap showing differentially expressed genes, based on relative Z-score, in the hippocampus of 7-month-old 5xFAD (AD) and 5xFAD/*Casp4*^*−/−*^ animals. **B** GO-biological process enrichment analysis and **C** WikiPathway enrichment analysis for the upregulated genes in 5xFAD *Casp4*^*−/−*^ versus 5xFAD animals. **D** GO-biological process enrichment analysis for genes upregulated in 5xFAD versus *Casp4*^*−/−*^ 5xFAD animals. **E** Volcano plot showing DEG (fold change > 1, *P* < 0.05) for 5xFAD/*Casp4*^*−/−*^ vs. 5xFAD mice. Heatmaps demonstrating differentially expressed genes: **F** neuroinflammation signaling pathway from IPA, **G** IL-8 signaling pathway from IPA, and **H** chemokine signaling pathway from KEGG

To define transcriptional underpinnings of the improved AD pathology in 5xFAD mice lacking CASP11, we performed bulk RNA-sequencing on the hippocampal region of 7-month-old age- and sex-matched 5xFAD and 5xFAD/*Casp4*^*−/−*^ mice. We identified ~ 860 differentially expressed genes among 5xFAD/*Casp4*^*−/−*^* versus* 5xFAD brains (Fig. 4A, Supplementary data file 2). This data is shared through Gene Expression Omnibus with accession number GSE227193. The upregulated genes in 5xFAD/*Casp4*^*−/−*^ brains were significantly enriched in various mitochondrial and metabolism-related pathways, such as electron transport chain and oxidative phosphorylation (Fig. 4B–C). In contrast, downregulated genes in the 5xFAD/*Casp4*^*−/−*^ brains compared to 5xFAD brains were enriched in cell–cell adhesion and autophagy molecules (Fig. 4D). The RNAseq data is also presented in a volcano plot with top genes highlighted for both upregulated (red) and downregulated genes (blue) in 5xFAD/*Casp4*^*−/−*^ brains compared to 5xFAD brains (Fig. 4E). Additionally, we highlight that known microglia homeostatic markers (genes *Cx3cr1*, *P2ry12*, *Hexb*, *Tmem119*) are not differentially expressed between the two sample sets.

Given the role of CASP11 in regulating inflammation and chemokine signaling, we further explored transcriptional changes in genes related to these pathways and indeed found multiple differentially expressed genes among the hippocampi of 5xFAD and 5xFAD/*Casp4*^*−/−*^ mice within the neuroinflammation signaling, IL-8 signaling, and chemokine signaling pathways (Fig. 4F–H). This led us to explore a role for CASP11 in inflammasome activation and IL-1β release in vitro.

As our male 5xFAD mice lacking CASP11 had dampened Aβ deposition, we also analyzed the data based on sex. For each group, only few genes that were differentially expressed between males and females were identified (Supplementary Table 1). The majority of these genes have previously been described as sexually dimorphic in the mouse hippocampus (*Xist*, *Eif2s3y*, *Uty*, *Ddx3y*, *Kdm5d*) [35]. There was one gene, *Ptgds* encoding prostaglandin D synthase, which is reduced in males compared to females in the 5xFAD mice. Differential expression of *Ptgds* between males and females was not apparent in 5xFAD/*Casp4*^*−/−*^ mice. Overall, we concluded that the involvement of CASP11 in driving neuroinflammation and Aβ production is influenced by sex; however, more analysis for the exact mechanism is required.

### Cytosolic delivery of fibrillar-Aβ promotes maturation and release of IL-1β from immune cells without cell death

**Fig. 5:**
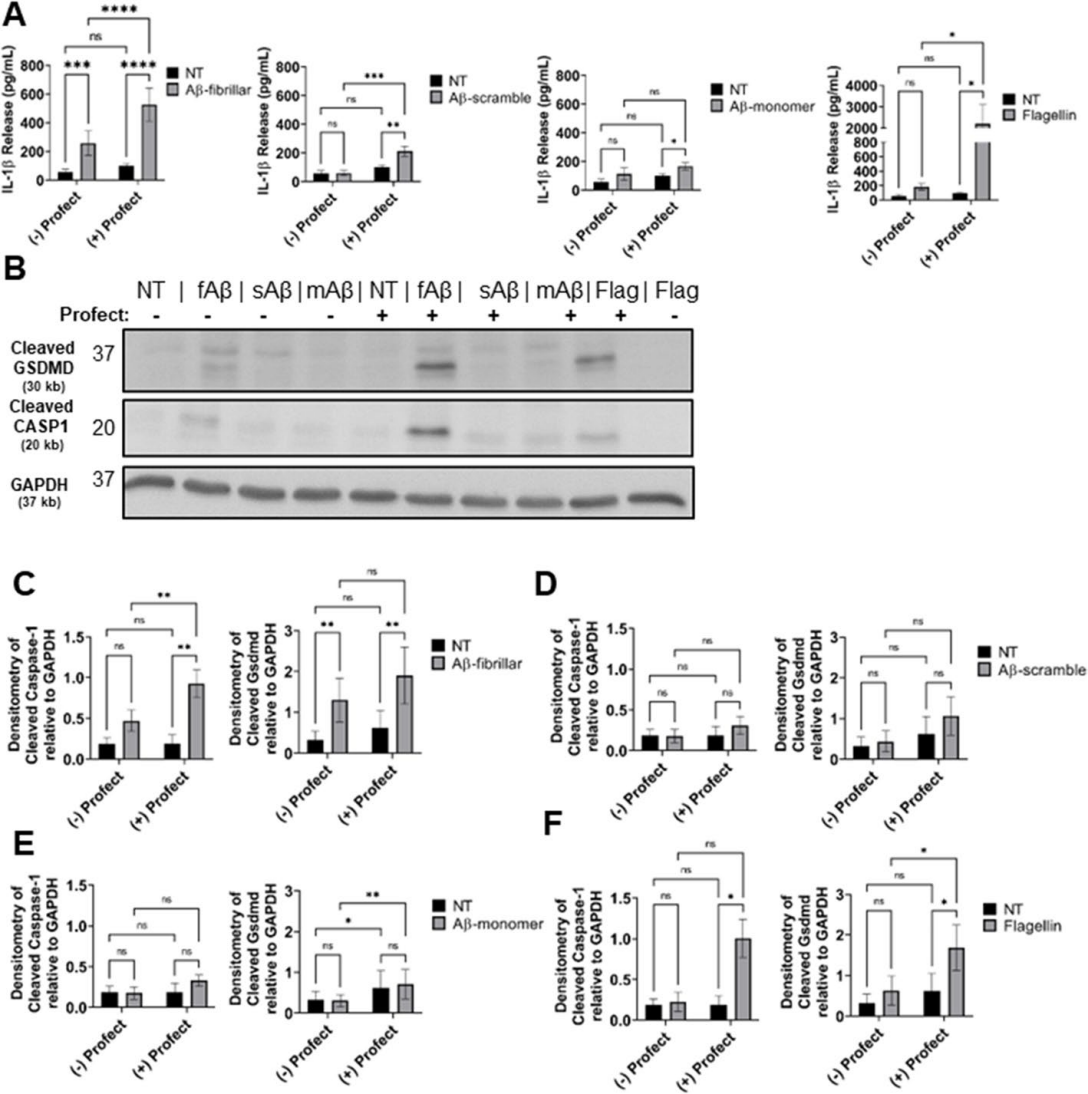
Profect-conjugated-fibrillar-Aβ (1–42) stimulates release of IL-1β and cleavage of Gasdermin-D and Caspase-1. **A** IL-1β release from LPS-primed mouse macrophages treated for 3 h with 10-µM fibrillar-Aβ (fAβ), 10-µM scramble-Aβ (sAβ), 10-µM monomer-Aβ (mAβ), and 250 ng/mL flagellin control or not treated (NT) with and without conjugation to cytosolic delivery reagent Profect (*N* = 10). **B** Representative immunoblots for cleaved GSDMD (30 kb), cleaved CASP1 (20 kb), and GAPDH (37 kb) from macrophages lysates treated as in **A**. **C**–**F** Densitometry analysis of immunoblots for cleaved GSDMD and cleaved CASP1 relative to GAPDH above background levels from LPS-primed mouse macrophages treated for 3 h with **C** 10-µM fibrillar-Aβ with and without Profect, **D** 10-µM scramble-Aβ with and without Profect, **E** 10-µM monomer-Aβ with and without Profect, and **F** 250 ng/mL flagellin with and without Profect (*N* = 6). Statistical analysis for **A** and **C**–**F** completed by two-way ANOVA Tukey’s multiple comparisons test with matching. For simplicity, graphs do not display *P*-values for all comparisons. *P ≤ 0.05, **P ≤ 0.01, ***P ≤ 0.001

Activation of the inflammasome occurs intracellularly where all components of the inflammasome machinery can interact upon activation [36]. Macrophages and microglia are phagocytic cells that can internalize and traffic Aβ to lysosomes, although microglia in the AD brain exhibit defects in lysosomal function [37]. The phagocytosis of increasing amounts of Aβ causes lysosomal rupture which results in release of the contents of the lysosome as well as cathepsin B into the cytosol [38, 39]. In this setting, cathepsin B serves as a signal for NLRP3 inflammasome activation. However, it remains unclear if the released Aβ in the cytosol could also activate the inflammasome as previous work did not differentiate the location of Aβ. Additionally, the contribution of CASP11 to IL-1β release in response to Aβ was not previously tested, and we saw that CASP11 promoted microglial release of IL-1β. Therefore, we utilized a system in which Aβ is primarily delivered to the cytosol of mouse macrophages. We conjugated various forms of Aβ(1–42) to Profect (Targeting Systems), a reagent which transports proteins directly across the cell membrane into the cytosol of eukaryotic cells. We pre-treated wild-type (WT) macrophages with LPS to prime pro-IL-1β gene expression [40–42]. Since soluble (monomeric), oligomeric, and fibrillar forms of Aβ contribute to AD pathology [43], primed macrophages were treated with fibrillar-Aβ, an amino acid scrambled-Aβ control, and monomer-Aβ all with or without conjugation to Profect. The conjugation to Profect significantly increased IL-1β release in response to fibrillar Aβ. We observed minimal, although significant, release of IL-1β in response to Profect-conjugated-monomeric or Profect-conjugated-scrambled-Aβ (Fig. 5A). Scrambled-Aβ and monomer-Aβ did not induce IL-1β release without Profect conjugation (Fig. 5A). To confirm that Profect successfully transported proteins into the cytosol, we used Profect-conjugated flagellin as a control [44]. Profect-conjugated flagellin stimulated significant release of IL-1β (Fig. 5A). These results indicate that fibrillar-Aβ promotes the release of IL-1β, and this effect is further enhanced when Aβ accesses the cytosol.

Previous work indicates that Aβ may prime the inflammasome through activation of toll-like receptors (TLRs) [42]. Therefore, we determined if fibrillar-Aβ(1–42) treatment would prime the inflammasome response in macrophages. Macrophages were treated with fibrillar-Aβ with or without conjugation to Profect. LPS was used as a positive control. We utilized ATP as the second-step activator [20]. LPS with ATP caused release of IL-1β; however, ATP treatment of macrophages primed with fibrillar-Aβ with or without conjugation to Profect did not release IL-1β (Supplementary Fig. 2A). This result indicates that fibrillar-Aβ(1–42) does not prime the inflammasome response in macrophages.

To show specifically that cytosolic fibrillar-Aβ activates the inflammasome response, we analyzed cell lysates for cleavage of CASP1 which is cleaved and activated by the inflammasome complex [45]. We found that CASP1 is cleaved in response to fibrillar-Aβ conjugated to Profect and flagellin conjugated to Profect, but not in response to any of the other forms of Aβ (Fig. 5B–F). Importantly, monomeric- and scrambled-Aβ conjugated to Profect did not cause CASP1 cleavage. Overall, these results indicate that cytosolic fibrillar-Aβ activates an inflammasome response leading to cleavage and activation of CASP1 and release of IL-1β.

The activation of CASP11 and/or CASP1 is accompanied by the cleavage of GSDMD and the release of the pore-forming fragment [46]. Cleaved GSDMD forms pores within the cellular plasma membrane allowing for release of mature IL-1β [20, 47]. To further determine the mechanism by which fibrillar-Aβ promotes IL-1β release, we analyzed cell lysates of WT macrophages and found that GSDMD is cleaved in response to fibrillar-Aβ, fibrillar-Aβ conjugated to Profect, and flagellin conjugated to Profect, but not in response to any of the other forms of Aβ with or without Profect (Fig. 5B–F). Typically, the excessive formation of GSDMD pores can lead to pyroptotic cell death accompanied by the release of high amounts IL-1β and lactate dehydrogenase (LDH) enzyme in some contexts [23, 48, 49]. However, under physiological conditions, the formation of few GSDMD pores in the membranes of afflicted cells is followed by membrane repair preventing the release of LDH and the occurrence of cell death [23, 50]. To deter

mine whether pyroptotic cell death was occurring in response to fibrillar-Aβ, the release of LDH into the culture supernatants was analyzed. There was no difference in LDH release between no treatment (NT) and fibrillar-Aβ with and without Profect conjugation (Supplementary Fig. 2B). Therefore, the cleavage of GSDMD and formation of pores in response to Aβ is not accompanied by cell death in response to fibrillar-Aβ.

### GSDMD is required for CASP11- and CASP1-mediated release of IL-1β following stimulation with fibrillar-Aβ

Earlier work suggests that the NLRP3 inflammasome mediates IL-1β release in response to Aβ, though the contribution of CASP11 to this process remains undetermined [38]. We first evaluated if Profect conjugated-Aβ stimulates IL-1β release in an NLRP3-dependent manner in macrophages. We primed wild-type (WT) and *nlrp3*^*−/−*^ macrophages with LPS and then treated with fibrillar-Aβ with or without conjugation to Profect. In response to fibrillar-Aβ conjugated to Profect, significantly reduced release of IL-1β in *nlrp3*^*−/−*^ macrophages was observed (Supplementary Fig. 3A). These results indicate that cytosolic fibrillar-Aβ promotes the release of IL-1β via an NLRP3-dependent mechanism. Of note, the expression of inflammasome components including NLRP3, CASP11, adapter protein apoptosis-associated speck-like protein containing a CARD (ASC), and pro-IL-1β did not significantly differ among WT and knockout macrophages (Supplementary Fig. 3B).

**Fig. 6:**
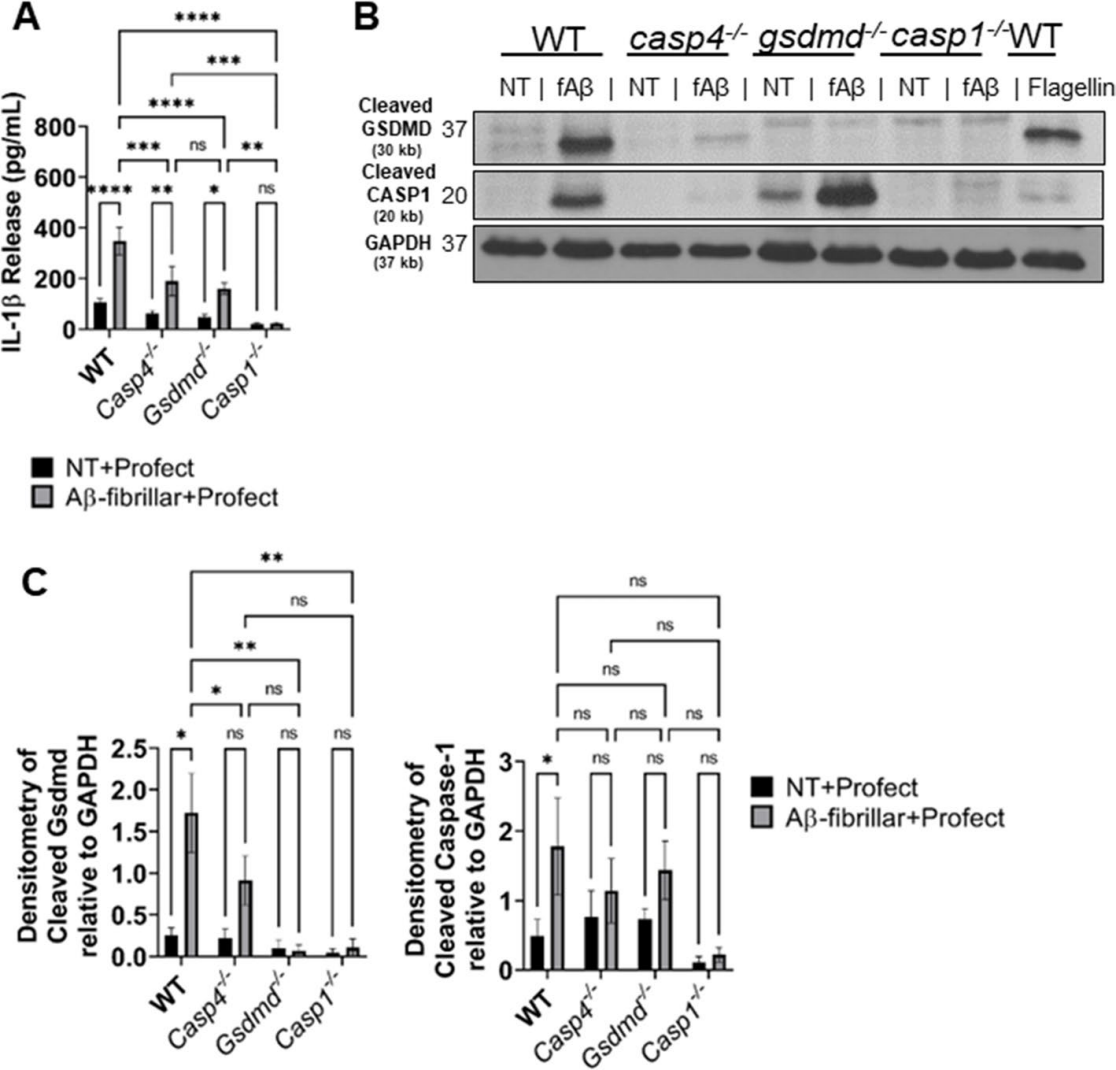
CASP11 promotes IL-1β release in response to Aβ by facilitating cleavage of GSDMD and CASP1. **A** IL-1β release from LPS-primed macrophages from wild-type (WT), *casp4*^*−/−*^, *gsdmd*^*−/−*^, and *casp1*^*−/−*^ mice treated for 3 h with 10-µM Profect-conjugated fibrillar-Aβ or Profect alone (NT + Profect) (*N* = 9). **B** Representative immunoblots for cleaved GSDMD (30 kb), cleaved CASP1 (20 kb), and GAPDH (37 kb) from macrophages lysates treated with Profect-conjugated fibrillar-Aβ (fAβ) or Profect alone (NT) as in **A**. **C** Densitometry analysis of immunoblots for cleaved GSDMD and cleaved CASP1 relative to GAPDH above background levels for macrophages treated as in **A** and **B** (*N* = 8 for WT and *casp4*^*−/−*^, *N* = 3 for *gsdmd*^*−/−*^, and *casp1*.^*−/−*^). Statistical analysis for **A** and **C** completed by two-way ANOVA Tukey’s multiple comparisons test. For simplicity, graphs do not display *P*-values for all comparisons. *P ≤ 0.05, **P ≤ 0.01, ***P ≤ 0.001, ****P ≤ 0.0001

In addition to the canonical NLRP3 inflammasome, the noncanonical inflammasome can also activate CASP1 through CASP11. CASP11 cleaves GSDMD in some settings allowing for the release of IL-1β in CASP1-dependent and independent manners [20]. However, the role of CASP11 in the inflammasome response to Aβ has not yet been differentiated. To determine if CASP11 mediates inflammasome activation in response to Aβ, we primed WT, *Casp4*^*−/−*^, *Gsdmd*^*−/−*^, and *Casp1*^*−*^ macrophages with LPS and then treated with fibrillar-Aβ with and without conjugation to Profect. In response to fibrillar-Aβ conjugated to Profect, significantly reduced levels of IL-1β were released from *Casp4*^*−/−*^ and *Gsdmd*^*−/−*^ and no IL-1β release from *Casp1*^*−/−*^ macrophages when compared to WT (Fig. 6A). These results indicate that CASP11 and GSDMD cleavage plays a role in promoting IL-1β release in response to fibrillar-Aβ in a CASP1-dependent manner.

To evaluate how CASP11 promotes IL-1β release, we analyzed the cleavage of CASP1 and GSDMD in cell lysates from LPS-primed WT, *Casp4*^*−/−*^, *Gsdmd*^*−/−*^, and *Casp1*^*−/−*^ macrophages treated with fibrillar-Aβ conjugated to Profect. CASP1 cleavage is significantly reduced in response to fibrillar-Aβ in *Casp4*^*−/−*^ macrophages (Fig. 6B–C), indicating that CASP11 expression promotes cleavage of CASP1. GSDMD can be cleaved by active CASP1 and/or CASP11 according to the insult [20]. To determine if CASP11 and/or CASP1 are upstream of GSDMD cleavage, we analyzed cleavage of GSDMD in the absence and presence of CASP11. We found that GSDMD cleavage was decreased in both *Casp4*^*−/−*^ and *Casp1*^*−/−*^ macrophages in comparison to WT (Fig. 6B–C), indicating that both CASP11 and CASP1 promote GSDMD cleavage in response to fibrillar Aβ.

To determine the role of CASP11 in inflammasome pathway activation, we analyzed the expression of components of the inflammasome pathway in the hippocampus of 5xFAD and 5xFAD/*Casp4*^*−/−*^ mice. There was no significant difference in expression of CASP1, cleaved CASP1, ASC, or pro-IL1β by Western blot (Supplementary Fig. 4). GSDMD and NLRP3 were not readily detected. These findings indicate that CASP11 does not alter the expression of CASP1, ASC, or pro-IL1β in the hippocampus of 5xFAD mice. Overall, these results indicate that both the noncanonical inflammasome and NLRP3 inflammasome mediate IL-1β release following exposure to Aβ via GSDMD.

## Discussion

In this study, we found that CASP4 (CASP11) is a driver of chronic neuroinflammation in AD in response to Aβ. Our work identified that CASP4 (CASP11) is upregulated in the temporal lobe (Brodmann area 38) of AD patients compared to age- and sex-matched non-dementia controls. According to a previous study which analyzed expression data from over a thousand AD patient samples, *CASP4* gene expression is also increased in the cerebellum, dorsolateral prefrontal cortex, and visual cortex of AD patients [24]. Notably, *CASP4* expression positively correlates with increased expression of *CASP1*, as well as AD risk factor genes *TREM2*, *CR1*, and *TYROBP* which are implicated in microglia-mediated inflammation [24]. An additional study found that *CASP4* expression is increased in the hippocampus of AD patients and correlates with clinical disease progression [25]. Our work indicates that epigenetic mechanisms contribute to increased expression of *CASP4* in the AD brain. We also discerned the contribution of members of the noncanonical inflammasomes to IL-1β release in response to Aβ. Additionally, we demonstrated that targeting *CASP4* in AD can alleviate Aβ accumulation and neuroinflammation.

In the AD field, several studies have completed analysis of the methylation status of specific candidate genes that are implicated in AD pathogenesis [51]. These include *APOE*, *BDNF*, *TREM2*, and glycogen synthase kinase 3 beta (*GSK3β*). Genome-wide DNA methylation studies on AD patients indicated that numerous pathways such as neurogenesis, amyloid generation, and inflammatory responses are implicated [51]. It is currently unclear whether key genes implicated in AD pathogenesis are associated with hypermethylation, hypomethylation, or a mix of both. According to our data, effectors involved in Aβ generation may experience global hypomethylation leading to increased production of Aβ which is in agreement with prior studies [51]. We also found that other critical neuropathways, such as cell adhesion and glutamate signaling, undergo hypermethylation as reported previously [51]. Although globally altering the methylation status was previously suggested as a therapeutic option in AD, these recent combined data suggest that benefits may be counteracted by unfavorable effects.

The precise mechanism underlying differential methylation of *CASP4* in AD patients remains unknown and warrants further evaluation. DNA methylation, denoted as 5-methylcytosine (5mC), is a well-characterized and highly stable epigenetic mechanism [52–54]. Differentiating mechanisms underlying demethylation programming has been crucial to understand this epigenetic mark of DNA hypomethylation [7]. Potential pathways include loss of maintenance DNA methylation programs, termed passive demethylation, as well as nucleotide and base excision repair pathways, or active demethylation [7, 8, 55]. DNA methyltransferase 1 (DNMT1) is responsible for maintenance of DNA methylation. Notably, DNMT1 and other methylation factors are decreased in Alzheimer’s disease brain tissue, which could lead to passive demethylation [8]. DNA demethylation can also be initiated by active mechanisms which include a mediator step of oxidation of 5mC into 5-hydroxymethylcytosine (5hmC) by the ten-eleven translocation (TET) proteins [7]. Notably, 5hmC is especially enriched in the brain [56]. Bilsufite sequencing as performed here converts both 5mC and 5hmC to uracil, thereby analyzing both as methylated sites [57]. In general, 5hmC is associated with increased gene activity, although this may depend on other factors [58]. A recent study found hundreds of differentially hydroxymethylated 5hmC regions associated with Aβ plaques and neurofibrillary tangles in human AD patients, yet *CASP4* was not identified as a differentially hydroxymethylated region [59]. According to our data, hypomethylation of DNA upstream of the *CASP4* transcription start site may stably enhance expression of CASP4 in the AD brain. Our data points to an epigenetic mechanism underlying the exacerbated IL-1β release and inflammasome activation seen in AD which improves our understanding of neuroinflammation in this disease. As CASP4 is primarily expressed in microglia, microglia-targeted analysis of DNA demethylation programs in AD would be beneficial to further understand the mechanism underlying increased *CASP4* expression. Accordingly, microglia-targeted methylation therapies can be effective and accompanied by less off target-mediated side effects.

We demonstrated by multiple methods that CASP11 protein is primarily expressed in microglia and is increased in 5xFAD microglia. In support of this finding, according to the online resource from the Barres group, *Casp4* mRNA is predominantly expressed in macrophages and microglia, with a lower level of expression in endothelial cells in healthy mouse brains [29, 30]. Microglia exhibit various roles in AD progression including generation of IL-1β, which drives production and seeding of Aβ plaques, exacerbates neurofibrillary tangle formation, and promotes tissue damage and synaptic dysfunction in AD [12–15, 38, 60, 61]. However, the mechanism underlying the elevated chronic production of IL-1β by microglia in AD and a potential role for CASP4 were still unclear.

We were able to analyze the contribution of CASP4 to AD disease progression by generating a 5xFAD mouse lacking murine *Casp4* expression. We found that CASP11 drives neuroinflammation and Aβ deposition. The RNAseq analysis on the hippocampus from 5 and 5xFAD/*Casp4*^*−/−*^ mice revealed differences in genes encoding important AD pathogenesis and neuroinflammatory regulators. We observed significantly decreased expression of *GSK3B* in 5xFAD/*Casp4*^*−/−*^ mice (Fig. 4A). *GSK3B* encodes glycogen synthase kinase 3β (GSK3β), which contributes to increased Aβ production, hyperphosphorylation of tau, and microglia activation [62]. Consequently, inhibition of GSK3β in mice reverses AD pathology [62]. We also observed a decrease in *MAPK1* which encodes mitogen-activated protein kinase 1 (MAPK1) in 5xFAD/*Casp4*^*−/−*^ (Fig. 4E–G). The MAPK family are crucial regulators of neuroinflammatory processes including generation of inflammatory cytokines [63]. Inhibition of MAPK1 in a rat model of AD improved cognitive function [64]. Our extensive work in CASP4 biology demonstrated consistently that CASP4 promotes inflammatory cell migration in various contexts [65, 66]. Prior work also demonstrated that expression of CASP4 promotes microglia clustering around Aβ plaques and increased inflammation in the brain of AD mice [24]. It is therefore likely that CASP4 promotes increased immune cell migration and activation in AD brains.

Reports have characterized IL-1β activation in response to Aβ [38, 61, 67], but our study evaluated the role of CASP11 for the first time as a new molecular player in the highly complex multi-protein process required for IL-1β activation and release in AD. Activation of the inflammasome, which promotes maturation and release of IL-1β, requires a priming signal and an activating signal [65]. The activating signal is a cytosolic pathogen-associated molecular pattern (PAMP) or a danger-associated molecular pattern (DAMP) [42]. CASP11 is known to potentiate inflammasome responses leading to GSDMD pore formation which drives the release of active cytokines [68–70]. Several studies demonstrated that NLRP3 promotes pathology in a mouse model of AD [61]. We demonstrate that CASP4 promotes cleavage of both CASP1 and GSDMD in vitro in macrophages treated with Profect-conjugated fibrillar Aβ. Currently, the mechanism of Aβ-induced inflammasome activation can be explained through several possibilities. It has been shown that Aβ promotes lysosomal damage leading to release of cathepsin B and subsequent activation of the NLRP3 inflammasome [38]. Notably, here we demonstrated that cytosolic Aβ directly stimulates an inflammasome response. It is also plausible that ROS production associated with Aβ phagocytosis activates the NLRP3 inflammasome [38, 39, 67]. Hence, precise mechanism of the interaction of Aβ with the canonical inflammasome requires further investigation.

IL-1β is known to stimulate the production of *amyloid precursor protein* (*APP*) and enhance the activity of enzymes that generate Aβ [12, 13]. Therefore, CASP4 may promote Aβ plaque generation by enhancing IL-1β production and release. Additionally, the release of apoptosis-associated speck-like protein containing a CARD (ASC) as a by-product of inflammasome activation serves as a seeding point for the formation of Aβ plaques [71]. Aβ bound to ASC is also more toxic to microglia and promotes increased microglia death and dysfunction [72]. CASP4 increases the release of ASC through GSDMD pores which allows for more Aβ plaques to form, thereby promoting continued activation of microglia. It is also possible that CASP4 alters the ability of microglia to clear Aβ via modulation of actin dynamics as observed in response to bacterial infection of macrophages [73–76]. Reduced Aβ burden in 5xFAD mice lacking CASP4 may therefore be due to reduced inflammation which reduces the production of Aβ by neuronal cells.

CASP4 expression is likely regulated in part by methylation status. Modulation of epigenetic mechanisms has been suggested as a potential strategy for the treatment of AD, especially with the success of this approach in some cancers [77–79]. Studies testing therapeutic DNA-methylating and DNA-demethylating agents for AD could utilize CASP4 expression as an epigenetic marker of microglia activity. In conclusion, we provide mechanistic evidence that CASP4 is a regulator of neuroinflammation in AD with potential as a therapeutic target.

## Materials and methods

### Human samples from NIH BioBank

Human temporal lobe brain tissue sections (Brodmann area 38) with AD and age-matched controls without dementia (non-dementia, ND) were obtained from the NIH BioBank in accordance with our institution’s IRB. These snap-frozen tissue samples were used for all TRIzol homogenization, RT-qPCR, and immunoblot analysis. Range of hours until freeze, age, race, and sex are in Supplementary Table 2.

### Reduced representation bisulfite sequencing and analysis

Five samples from AD and age-matched non-dementia controls were prepared for RRBS analysis. DNA methylation data were generated for all samples using the reduced representation bisulfite sequencing (RRBS) method [80]. Briefly, NEXTFLEX Bisulfite Library Prep Kit for Illumina Sequencing (Bioo Scientific, PerkinElmer, MA, USA) was used to convert the study sample genomes to bisulfite-converted genomes. Illumina sequencing libraries were prepared using Zymo EZ DNA methylation kit. Bisulfite converted libraries were sequenced to 35–40 million PE-150 × 150bp clusters per sample using HiSeq 4000 Sequencer (Illumina, CA, USA).

Raw read QC assessed with FastQC v0.11.5 before trimming and after trimming. Trimming was performed with TrimGalore v0.4.5 specifying RRBS and Illumina with a quality cutoff of 20. Quality and adapter trimmed reads aligned to bismark generated indexes for GRCm38 with bismark v0.22.1 using bowtie2 v2.3.5.1 with options “ –N 1 –L 15 -D 50 –score_min L,-0.6,-0.6 -p 10 –X 600” methylations extracted using bismark_methylation_extractor. Reports generated with bismark2report. Alignment bam sorted and indexed with samtools v1.9. Differential methylation of regions performed with R package DSS running DMLtest with group 1 as control and group 2 as Alzheimer’s and callDML and callDMR with option p.threshold = 0.05. Regions annotated with R package AnnotationDbi and human database org.Hs.eg.db.

DMR results filtered with a threshold of 50% change in methylation ratio and *P-value* < *0.01*, with > 5 reads in at least two samples among human brain samples. Gene Ontology biological process enrichment analysis was performed using Enrichr for genes with hypomethylated or hypermethylated DMRs [81].

### Targeted DNA methylation analysis of the CASP4 locus

Genomic DNA was extracted from snap-frozen brain samples using the PureLink® Genomic DNA Mini Kit (cat no.: K182002, Invitrogen) and then bisulfite treated using the EZ DNA Methylation-Direct Kit (Zymo) to convert all unmethylated cytosines to uracil. Locus-specific PCR was performed on the bisulfite-converted DNA using the primers specific for the DMR at the *CASP4* locus: *FP* (*ggaatttagtttttgatttggggg*) and *RP* (*cccacctaaaaaaacaatctaacc*). The amplicon DNA size was confirmed by gel electrophoresis and purified using the Zymoclean Gel DNA Recovery Kit (Zymo). DNA sequencing library of the purified CASP4 PCR amplicons was prepared using the Native Barcoding Kit (cat. no.: EXP-NBD104) and Ligation Sequencing Kit (cat. no.: SQK-LSK109) (Oxford Nanopore Technologies). Sequencing was performed on the prepared library using an R9 flow cell and MinION device (Oxford Nanopore Technologies). The resultant FASTQ files from the MinION sequencing were extracted for downstream analysis of CpG methylation at the amplified DMRs. In brief, the FASTQ files were quality controlled by first removing the multiplexing barcodes using Porechop v0.2.4 (github.com/rrwick/Porechop) with the default parameters and then filtering by read quality (-q 10) and length (-l 200) using NanoFilt v2.6.0. [82]. The quality-controlled reads were then mapped against the PCR target amplicons (-x map-ont) using minimap2 v2.17-r941 [83]. For each amplicon, the different types of bases mapped at each position in the bam files (count –bases -w 1) were counted using igvtools v2.8.0 [84]. The CpG sites were automatically extracted from the PCR target sequences (locate -i -P -p cg) using SeqKit v0.12.0 [85], and custom bash scripts were used to extract the base distribution from igvtools at these specific CpG locations and tabulate them per amplicon.

### Mice

C57BL/6 wild-type (WT) mice and 5xFAD mice (stock no. 034840) were obtained from the Jackson Laboratory (Bar Harbor, ME, USA). C57BL/6 WT mice were obtained from the Jackson Laboratory (Bar Harbor, ME, USA). *Casp4*^*−/−*^ mice were generously provided by Dr. Yuan at Harvard Medical School, Boston, MA, USA, and are now available from the Jackson Laboratory (024698) [18]. *Casp-1*^*−*/*−*Casp11Tg^ mice were kindly provided by Dr. Vishva Dixit at Genentech, San Francisco, CA, USA. *Gsdmd*^*−/−*^ mice and *nlrp3*^*−/−*^ bones were provided by Dr. Thirumala-Devi Kanneganti at St. Jude Children’s Research Hospital, Memphis, TN, USA. All mice were housed in a pathogen-free facility, and experiments were conducted with approval from the Animal Care and Use Committee at the Ohio State University (Columbus, OH, USA).

### Generation of 5xFAD/Casp4 strain

The 5xFAD (AD) (B6SJL-Tg (APPSwFlLon, PSEN1 × M146L × L286V) 6799Vas/Mmjax) mouse is a double transgenic APP/PS1 mouse model that co-expresses five AD mutations leading to accelerated plaque formation and increased Aβ42 production. AD mouse model over-expresses APP with K670N/M671L (Swedish mutation), I716V (Florida mutation), and V717I (London mutation), PS1 with M146L, and L286V mutations. These mice accumulate high levels of intraneuronal Aβ-42 around 1.5 months of age with amyloid deposition around 2 months [28, 86]. The 5xFAD mice were crossed with *Casp4*^*−/−*^ mice for 5–6 generations for use in tissue or behavioral assessments. 5xFAD mice were maintained hemizygous by crossing littermate mice not carrying the transgene with those carrying one copy of the transgene in order to generate the full *Casp4* knockout. Genotyping was carried out for *Casp4* mutant and 5xFAD transgene as specified by the Jackson Laboratory.

### Preparation of mouse hippocampus

Mouse brains were dissected following euthanasia by CO_2_. The entire hippocampus was immediately dissected on ice as described previously [87] and snap frozen in liquid nitrogen and stored at − 80 °C. The hippocampus was cryopulverized into a fine powder on a liquid-nitrogen frozen BioPulverizer (cat no.: 59012MS, BioSpec). The powder was mixed well and collected in separate tubes for future analysis. For immunoblot and RT-qPCR analysis, brains were lysed in TRIzol. For RNAseq analysis, brains were lysed as with lysis buffer from PureLink™ RNA Mini Kit (Invitrogen, 012183025) by manufacturer’s instructions.

### Microglia isolation

A 5-month-old, sex-matched 5xFAD and WTAD mice were utilized for microglia isolation to analyze expression of CASP11 by immunoblot. Microglia were isolated by MACS neural dissociation kit (Miltenyi Biotec, 130–107-677) followed by CD11b magnetic bead (Miltenyi Biotec, 130–093-634) isolation technique to positively select for brain microglia expressing the pan-microglia marker CD11b as has been described before [31, 88]. Microglia (CD11b +) and non-microglia (CD11b −) fractions were collected. Cells were pelleted and washed one time with PBS prior to lysing with TRIzol.

### Cell culture

Primary bone marrow-derived macrophages were derived from mice as previously described [75]. Briefly, tibias and femurs were flushed with IMDM media (Thermo Fisher Scientific, 12440053) supplemented with 10% heat inactivated fetal bovine serum (FBS, Thermo Fisher Scientific, 16000044), 50% L cell-conditioned media, 0.6 × MEM Non-Essential Amino Acids (Thermo Fisher Scientific, 11140050), and 0.1% penicillin and streptomycin (Thermo Fisher Scientific, 15140122). Cells were cultivated at least 6 days at 37 °C in a humidified atmosphere containing 5% CO_2_.

Microglia were isolated from the brains of 8- to 10-month-old mice and then cultured in the same media listed for macrophages. For measurement of IL-1β in cell culture supernatant, microglia were primed with 100 ng/mL LPS for 3 h, and then supernatant was collected after 24 h of culture.

### Aβ peptides and fibrillization

Aβ peptides in powder form (beta-amyloid (1–42) cat. no.: AS-20276; scrambled-beta-amyloid (1–42) cat. no.: AS-25382, AnaSpec) were initially resuspended in 1% NH_4_OH (ca.t no.: AS-61322, AnaSpec) for 15 min to dissolve any pre-formed aggregates per manufacturer instructions and then diluted to a final concentration of 0.05% NH_4_OH in water prior to fibrillization. Aβ peptides were converted to the fibrillar form of Aβ by incubating monomeric human Aβ(1–42) at 220 μM in water at 37 °C for 3 days prior to use as previously described [89]. Scrambled Aβ was also treated for fibrillization, although this should not form aggregates.

### in vitro treatment of primary macrophage

Macrophages were cultivated in IMDM media supplemented with 10% FBS at 1 × 10^6^ cells per well in a 12-well plate. Macrophages were primed for 3 h with 100 ng/mL Ultrapure LPS, *Escherichia coli* 0111:B4 (InvivoGen). A 10-µM Aβ peptides were conjugated to Profect-P1-lipid-based protein delivery reagent (cat no.: 0041, Targeting Systems) in 100-µL serum-free RPMI for 20 min prior to gentle resuspension in a final volume of 425 µL. The concentration of 10-µM Aβ peptides was selected based on prior publications studying the role of Aβ in intracellular inflammasome activation [38, 72, 90, 91]. Negative control Profect was incubated without any Aβ peptide. Macrophages were washed three times with serum-free RPMI to remove serum and free LPS prior to treatment with Profect complexes. Macrophages were treated with 400-µL Profect complexes for 3 h. Supernatant was collected for analysis by ELISA, and macrophages were lysed in TRIzol reagent. For experiments utilizing ATP, macrophages were not primed with LPS and instead treated first with Profect complexes for 3 h or LPS as a positive control and then with or without 5 mM ATP (cat no.: A6419, Sigma-Aldrich) for 30 min.

### Immunoblot

Protein extraction was performed using TRIzol reagent (Thermo Fisher Scientific) according to the manufacturer’s instructions. Briefly, after phase separation using chloroform, 100% ethanol was added to the interphase/phenol–chloroform layer to precipitate genomic DNA. Subsequently, the phenol-ethanol supernatant was mixed with isopropanol to isolate proteins. The Bradford method was used to determine protein concentrations. Equal amounts of protein were separated by 13.5% SDS-PAGE and transferred to a polyvinylidene fluoride (PVDF) membrane. Membranes were incubated overnight with antibodies against human CASP4 (cat. no.: M029-3, MBL), mouse Caspase-11 (cat. no.: NB120-10454, Novus Biologicals), human Aβ (D54D2) (cat. no.: 8243S, Cell Signaling Technology), mouse Gasdermin-D (cat. no.: ab209845, Abcam), mouse Caspase-1 (cat. no.: AG-20B-0042-C100, AdipoGen), ASC (cat. no.: 67824, Cell Signaling Technology), NLRP3 (cat. no.: 15101, Cell Signaling Technology), IL1β (cat. no.: GTX74034, Genetex), β-actin (cat. no.: 3700S, Cell Signaling Technology), or GAPDH (cat. no.: 2118, Cell Signaling Technology). Corresponding secondary antibodies conjugated with horseradish peroxidase (cat. no.: 7074 Rabbit, cat. no.: 7076 Mouse, cat. no.: 7077 Rat, Cell Signaling Technology) in combination with enhanced chemiluminescence reagent (cat. no.: RPN2209, Amersham) were used to visualize protein bands. Densitometry analyses were performed by normalizing target protein bands to their respective loading control using ImageJ software as previously described [73]. Full blot images at different exposures are provided in Supplementary Fig. 5.

### Measurement of IL-1β

The level of IL-1β in macrophage culture supernatants was measured by R&D Systems DuoSet ELISA Development Systems (murine IL-1b, DY401) according to the manufacturer’s instructions. The level of IL-1β in microglia culture supernatants was measured by ProcartaPlex Mouse Cytokine and Chemokine kit (cat. no.: EPXR260-26088–901, Thermo Fisher Scientific) using the MAGPIX plate reader according to the manufacturer’s instructions.

### LDH release

LDH release from macrophages treated with Aβ Profect complexes was measured using the CytoTox-ONE Homogeneous Membrane Integrity Assay (cat. no.: G7891, Promega) according to the manufacturer’s instructions. Aβ-induced LDH release [%] = ((test sample)/(high control)) × 100.

### Quantitative real-time PCR (RT-qPCR)

Total RNA was isolated from cryopulverized tissue lysed in TRIzol (cat. no.: 15596026; Invitrogen Life Technologies). Chloroform (cat. no.: 268320010, Fisher Scientific), isopropanol (cat. no.: BP2618212, Fisher Scientific), and glycogen (cat. no.: 10814010, Fisher Scientific) were used to isolate total RNA, and its concentration was measured by NanoDrop. The expression of genes was determined as previously described and expressed as relative copy numbers (RCN) [92]. RCNs are relative to housekeeping genes GAPDH and CAP-1 and multiplied by a factor of 100. Ct values of each *gene* were subtracted from the average Ct of the internal control. The resulting ΔCt was used in the equation: *RCN* = (2^−ΔCt^) × 100. The following primer sets were used: Human *CASP4* (FP-cacaacgtgtcctggagaga, RP-acttcctctaggtggcagca), human *CASP5* (FP-agtcagtgctgagggcattt, RP-ccctctaggatgccatgaga), and mouse *Caspase-4* (FP-catcactagactcatttcctgctt, RP-ctggaatttcaggaatagaatgtg).

### RNA-sequencing and data analysis

Total RNA was extracted from cryopulverized hippocampal tissue by PureLink™ RNA Mini Kit (Invitrogen, 012183025) according to the manufacturer’s instructions following lysis with the provided lysis buffer. RNA cleaning and concentration were done using Zymo Research, RNA Clean, and Concentrator™-5 kit (cat. no. R1015) following the manufacturer’s protocol. Fluorometric quantification of RNA and RNA integrity analysis was carried out using RNA Biochip and Qubit RNA Fluorescence Dye (Invitrogen). cDNA libraries were generated using NEBNext® Ultra™ II Directional (stranded) RNA Library Prep Kit for Illumina (NEB no. E7760L). Ribosomal RNA was removed using NEBNext rRNA Depletion Kit (human, mouse, rat) (E no. E6310X). Libraries were indexed using NEBNext Multiplex Oligos for Illumina Unique Dual Index Primer Pairs (NEB no. 644OS/L). Library Prep generated cDNA was quantified and analyzed using Agilent DNA chip and Qubit DNA dye. Ribo-depleted total transcriptome libraries were sequenced on an Illumina NovaSeq SP flow cell (paired-end 100 × 100 bp format; 35–40 million clusters, equivalent to 70–80 million reads. Library preparation, QC, and sequencing were carried out at Nationwide Children’s Hospital Genomic Core.

RNA-sequencing data processing and analysis were performed by the Biomedical Informatics Shared Resource (BISR) group at the Ohio State University using previously published pipeline [93]. Briefly, raw reads were aligned to mouse reference genome GRCm38 with HISAT2 v2.1.0 [94]. Gene-wise counts were generated with featureCounts from the subread package v1.5.1 for genes annotated by ensembl Mus_musculus.GRCm38.102, counting the primary alignment in the case of multimapped reads [95]. Raw counts were normalized by voom [96]. Genes were included if at least half of the samples had an expression of 2 CPM. Heatmaps were plotted with R package ComplexHeatmap. DESeq2 rlog transformation was used to process count data for PCA plotting. Functional enrichment analysis was performed with Ingenuity Pathway Analysis to enrich for IPA canonical pathways and with clusterProfiler to enrich for KEGG and GO terms. We define FDRsig as *FDR* < 0.05 and psig as *p* < 0.05.

Partek Flow was used for differential gene expression analysis using gene-specific analysis (GSA) algorithm by applying multiple statistical models to each individual gene in order to account for each gene’s varying response to different experimental factors and differing data distribution. Differentially expressed genes (DEGs) were identified for genes showing fold change ≥ 1 and *P-value* < *0.05*. WikiPathway and Gene Ontology nonredundant biological process enrichment analyses of DEGs among mouse WT and CASP4-KO brain samples were performed using WebGestalt with mouse genome as a reference set [97].

UMAP plots for expression of CASP4 and CXCR3 were generated, as previously described [32], to show expression levels of indicated genes using snRNA-seq datasets from female mouse brain tissues of young and aged animals (GSE207848).

### Immunohistochemistry (IHC) for mouse tissues and confocal analysis

Mice were anesthetized using ketamine/xylazine mixture and perfused as described previously [31]. Briefly, the heart was surgically exposed, and a perfusion needle was inserted directly into the left ventricle. Perfusion needle was secured in the left ventricle by using a hemostat surgical clamp. An incision was made in the right atrium to create an open circulation. Heparinized sterile PBS was injected to flush the blood out of the circulation and was followed by the injection of 4% paraformaldehyde (PFA) fixative solution. Brains were dissected and then underwent post‐perfusion fixation overnight at 4 °C in 4% paraformaldehyde‐PBS. Brains were then transferred to 30% sucrose (w/v) ‐PBS. Brains were embedded in optimal cutting temperature compound (OCT) and sectioned using a cryostat. Cryosections of 15–20 μm thickness were mounted on glass slides and stained using antibodies or ISH probes.

Immunofluorescence (IF) staining of mouse brain sections has been performed as previously described [31, 98]. Slides were washed three times for 15 min with PBS to remove residual OCT. The sections were then incubated in the blocking solution (PBS containing 10% donkey serum (cat. no.: S30-100 ml, Millipore Sigma), 2% BSA (cat. no.: BP1600-100, Fisher Scientific), and 0.3% Triton X-100 (cat. no.: BP151-100, Fisher Scientific) for 2 h at room temperature. Sections were then transferred to blocking solution containing the primary antibody for Aβ (D54D2) (cat. no.: 8243S, Cell Signaling Technology) and incubated overnight at 4 °C. After that, sections were washed with PBS 3 × for 15 min each. Then, they were incubated with the blocking solution containing the secondary antibody (donkey anti-Rabbit IgG (cat. no.: A32790, ThermoFisher Scientific) for 2 h at room temperature. DAPI (cat. no.: D1306, Fisher Scientific) was added to the staining solution in the last 15 min of incubation at a final concentration (5 µg/ml). Finally, sections were washed with PBS 3 × for 15 min. Antifade mounting media (cat. no.: P36934, Thermo Fisher Scientific) was added before cover-slipped. Fluorescent images were captured on Olympus FV 3000 inverted microscope with a motorized stage using 60 × /1.4 NA oil objective. Images were taken at z-sections of 0.5-μm intervals by using the 488 nm, 543 nm, and 405 nm (for DAPI) lasers.

### Flow cytometric analysis of microglia

Mice were intravenously injected while alive with anti-CD45-PE (BD Biosciences) (3 µg in 100 µl of sterile PBS) to discriminate between intravascular myeloid cells and intraparenchymal myeloid cells [99]. After sacrifice, brains were collected and processed into a single-cell suspension using adult brain dissociation kit (Miltenyi Biotec, catalog no. 130–107-677) as previously described [31]. Cells were then washed with cold PBS before staining with live/dead Zombie NIR fixable viability dye (BioLegend) for 30 min at 4 °C. Cells were then washed twice with PBS supplemented with 1% heat-inactivated FBS (1% FBS) (FACS buffer) and resuspended in Fc Block (clone 93) (eBioscience) at 4 °C for 5 min before surface staining with a mixture of the following Abs for 20 min at 4 °C: anti-CD86-APC-R700 (BD Bioscience), anti-CD45- BUV737 (BD Biosciences), and anti-CD11B-APC (Thermo Scientific). Fluorescence minus 1 was used as a negative control. Samples were collected on a Cytek Aurora flow cytometer (Cytek Biosciences). Analysis was performed using FlowJo software version 10.8.0. Microglia were identified as CD45 intermediate/CD11B high cell population among live cells.

### Statistical analysis

All figures display mean ± standard error of the mean (SEM) from at least three independent experiments as indicated in the figure legends. Comparisons between groups were conducted with either two-sample *t*-test, two-way ANOVA with Tukey’s correction for multiple comparisons if needed, or mixed-effects model with Holm’s adjustment for multiple comparisons as indicated depending on the data structure. Adjusted *P* < 0.05 was considered statistically significant. Data were analyzed using GraphPad Prism 6.0 (*t*-test and ANOVA) and SAS 9.4 (linear mixed-effects models).

### Supplementary Information

**Additional file 1: Supplementary Figure 1.** Microglial cells express high levels of Casp4 within mouse brains. A) UMAP visualization of brain cell meta-clusters identified in single-nucleus RNA-sequencing analysis of female mouse brain tissues (GSE207848). UMAP plots showing high RNA expression levels of Casp4 (B) and Cx3cr1 (C) within microglial cells from mouse brain tissues. **Supplementary Figure 2.** Fibrillar Aβ(1-42) does not prime the inflammasome response or promote cell death. A) IL-1β release from resting macrophages treated for 3 hours with 10µM fibrillar-Aβ (fAβ) or not treated (NT) with and without conjugation to cytosolic delivery reagent Profect or with LPS control followed by 30-minute activation with 5mM ATP (*N*=4 or *N*=3 for LPS only). Statistical analysis completed by mixed effects analysis with Tukey’s multiple comparisons test. B) Cell death measured by % LDH release (relative to high control) from LPS-primed mouse macrophages treated for 3 hours with 10µM fibrillar-Aβ with and without Profect, Statistical analysis completed by 2way ANOVA Tukey’s multiple comparisons test (*N*=5). **Supplementary Figure 3.** The NLRP3 inflammasome promotes IL-1β release in response to Profect-conjugated-Fibrillar Aβ(1-42). A) IL-1β release from LPS-primed macrophages from wild-type (WT) and nlrp3-/- mice treated for 3 hours with 10µM fibrillar-Aβ or not treated (NT) with and without conjugation to Profect (*N*=3). B) Representative immunoblots for NLRP3, CASP11, ASC, Pro-IL1β and loading control GAPDH from macrophages lysates treated with Profect-conjugated fibrillar-Aβ (fAβ) or Profect alone (NT) as in Figure 6A (*N*=2). Statistical analysis completed by 2way ANOVA Tukey’s multiple comparisons test. For simplicity, graph does not display p-values for all comparisons. *P ≤ 0.05, **P ≤ 0.01. **Supplementary Figure 4.** Expression of inflammasome components in the hippocampus of 5xFAD and 5xFAD/Casp4-/- mice. Immunoblots for CASP1, cleaved CASP1, ASC, Pro-IL1β and GAPDH loading control in hippocampal lysate from 7-month-old littermate Casp4-/- and 5xFAD/Casp4-/- mice and corresponding densitometry above background graphs (*N*=4). Statistical analysis completed by student’s t-test. **Supplementary Figure 5.** Full immunoblot staining for figures as indicated. Exposure times may vary from figure to best represent full antibody staining.

**Additional file 2: Supplementary Table 1.** Overlap of gene lists with FDR<0.05 when comparing male vs. female mice in a single group. **Supplementary Table 2.** The patient demographic and clinical characteristics of the human samples used in the study. Samples used for methylation analysis are marked in the DMR column. Y: years, BA: Brodmann’s area, h: hours, M: male, F: female, W: White, H: Hispanic, AA: African American, CASP4 DMR: CASP4 differentially methylated region.

**Additional file 3: Supplementary Data 1.** Filtered DMR list from RRBS. **Supplementary Data 2.** RNA sequencing, AD/Casp4 KO vs WT.

## Data Availability

Methylation and RNA-sequencing data sets are available online as referenced in the text.
