# Supplementary figures and images for: DNA hypomethylation promotes the expression of CASPASE-4 which exacerbates inflammation and amyloid-β deposition in Alzheimer’s disease
Source: Alzheimers Res Ther. 2024 Feb 8;16:29. doi: 10.1186/s13195-024-01390-2 (PMC10851453; doi:10.1186/s13195-024-01390-2)

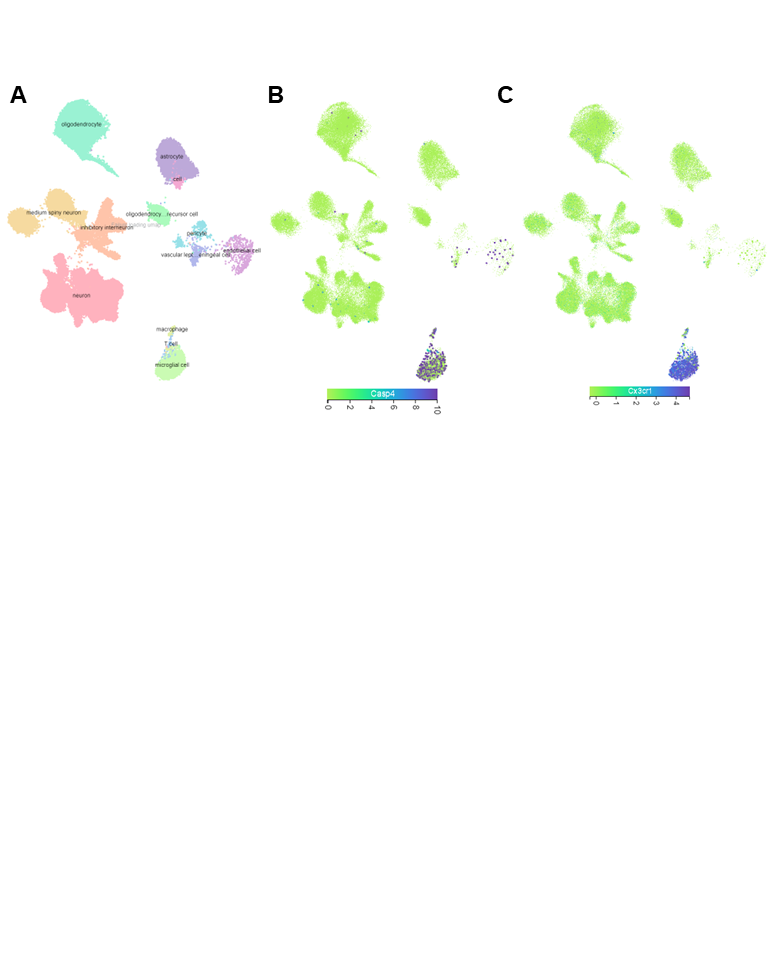

Supplement: Supplementary file 1 — Additional file 1: Supplementary Figure 1. Microglial cells express high levels of Casp4 within mouse brains. A) UMAP visualization of brain cell meta-clusters identified in single-nucleus RNA-sequencing analysis of female mouse brain tissues (GSE207848). UMAP plots showing high RNA expression levels of Casp4 (B) and Cx3cr1 (C) within microglial cells from mouse brain tissues. Supplementary Figure 2. Fibrillar Aβ(1-42) does not prime the inflammasome response or promote cell death. A) IL-1β release from resting macrophages treated for 3 hours with 10µM fibrillar-Aβ (fAβ) or not treated (NT) with and without conjugation to cytosolic delivery reagent Profect or with LPS control followed by 30-minute activation with 5mM ATP (N=4 or N=3 for LPS only). Statistical analysis completed by mixed effects analysis with Tukey’s multiple comparisons test. B) Cell death measured by % LDH release (relative to high control) from LPS-primed mouse macrophages treated for 3 hours with 10µM fibrillar-Aβ with and without Profect, Statistical analysis completed by 2way ANOVA Tukey’s multiple comparisons test (N=5). Supplementary Figure 3. The NLRP3 inflammasome promotes IL-1β release in response to Profect-conjugated-Fibrillar Aβ(1-42). A) IL-1β release from LPS-primed macrophages from wild-type (WT) and nlrp3-/- mice treated for 3 hours with 10µM fibrillar-Aβ or not treated (NT) with and without conjugation to Profect (N=3). B) Representative immunoblots for NLRP3, CASP11, ASC, Pro-IL1β and loading control GAPDH from macrophages lysates treated with Profect-conjugated fibrillar-Aβ (fAβ) or Profect alone (NT) as in Figure 6A (N=2). Statistical analysis completed by 2way ANOVA Tukey’s multiple comparisons test. For simplicity, graph does not display p-values for all comparisons. *P ≤ 0.05, **P ≤ 0.01. Supplementary Figure 4. Expression of inflammasome components in the hippocampus of 5xFAD and 5xFAD/Casp4-/- mice. Immunoblots for CASP1, cleaved CASP1, ASC, Pro-IL1β and GAPDH loading contr [file 13195_2024_1390_MOESM1_ESM.zip › Supplementary Figure 1.tif]

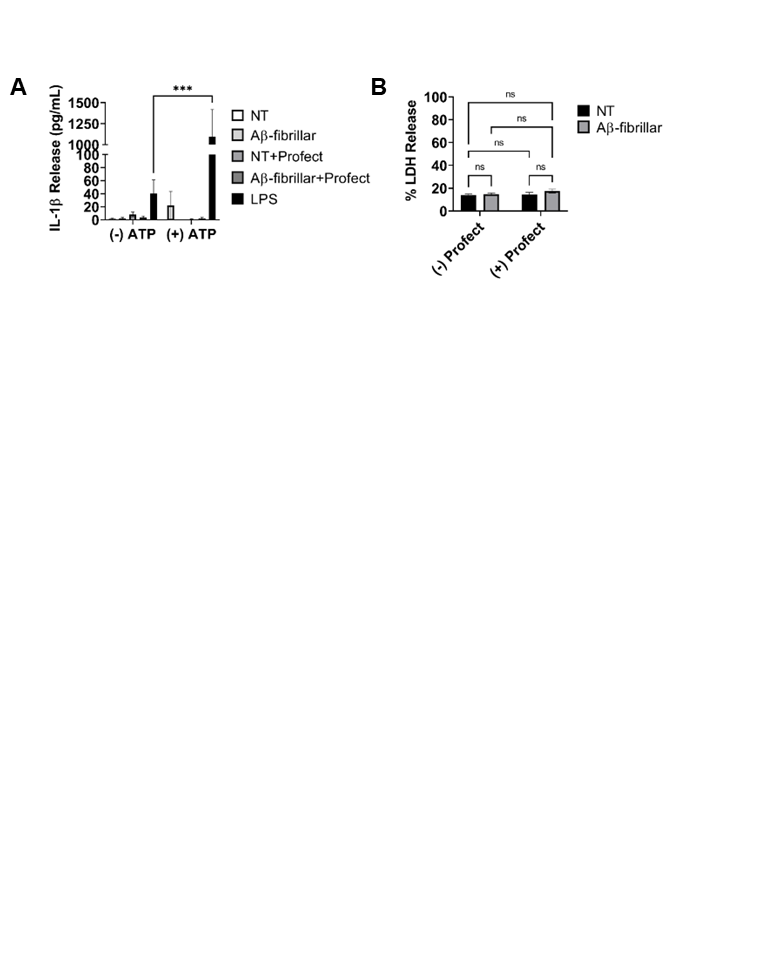

Supplement: Supplementary file 1 — Additional file 1: Supplementary Figure 1. Microglial cells express high levels of Casp4 within mouse brains. A) UMAP visualization of brain cell meta-clusters identified in single-nucleus RNA-sequencing analysis of female mouse brain tissues (GSE207848). UMAP plots showing high RNA expression levels of Casp4 (B) and Cx3cr1 (C) within microglial cells from mouse brain tissues. Supplementary Figure 2. Fibrillar Aβ(1-42) does not prime the inflammasome response or promote cell death. A) IL-1β release from resting macrophages treated for 3 hours with 10µM fibrillar-Aβ (fAβ) or not treated (NT) with and without conjugation to cytosolic delivery reagent Profect or with LPS control followed by 30-minute activation with 5mM ATP (N=4 or N=3 for LPS only). Statistical analysis completed by mixed effects analysis with Tukey’s multiple comparisons test. B) Cell death measured by % LDH release (relative to high control) from LPS-primed mouse macrophages treated for 3 hours with 10µM fibrillar-Aβ with and without Profect, Statistical analysis completed by 2way ANOVA Tukey’s multiple comparisons test (N=5). Supplementary Figure 3. The NLRP3 inflammasome promotes IL-1β release in response to Profect-conjugated-Fibrillar Aβ(1-42). A) IL-1β release from LPS-primed macrophages from wild-type (WT) and nlrp3-/- mice treated for 3 hours with 10µM fibrillar-Aβ or not treated (NT) with and without conjugation to Profect (N=3). B) Representative immunoblots for NLRP3, CASP11, ASC, Pro-IL1β and loading control GAPDH from macrophages lysates treated with Profect-conjugated fibrillar-Aβ (fAβ) or Profect alone (NT) as in Figure 6A (N=2). Statistical analysis completed by 2way ANOVA Tukey’s multiple comparisons test. For simplicity, graph does not display p-values for all comparisons. *P ≤ 0.05, **P ≤ 0.01. Supplementary Figure 4. Expression of inflammasome components in the hippocampus of 5xFAD and 5xFAD/Casp4-/- mice. Immunoblots for CASP1, cleaved CASP1, ASC, Pro-IL1β and GAPDH loading contr [file 13195_2024_1390_MOESM1_ESM.zip › Supplementary Figure 2.tif]

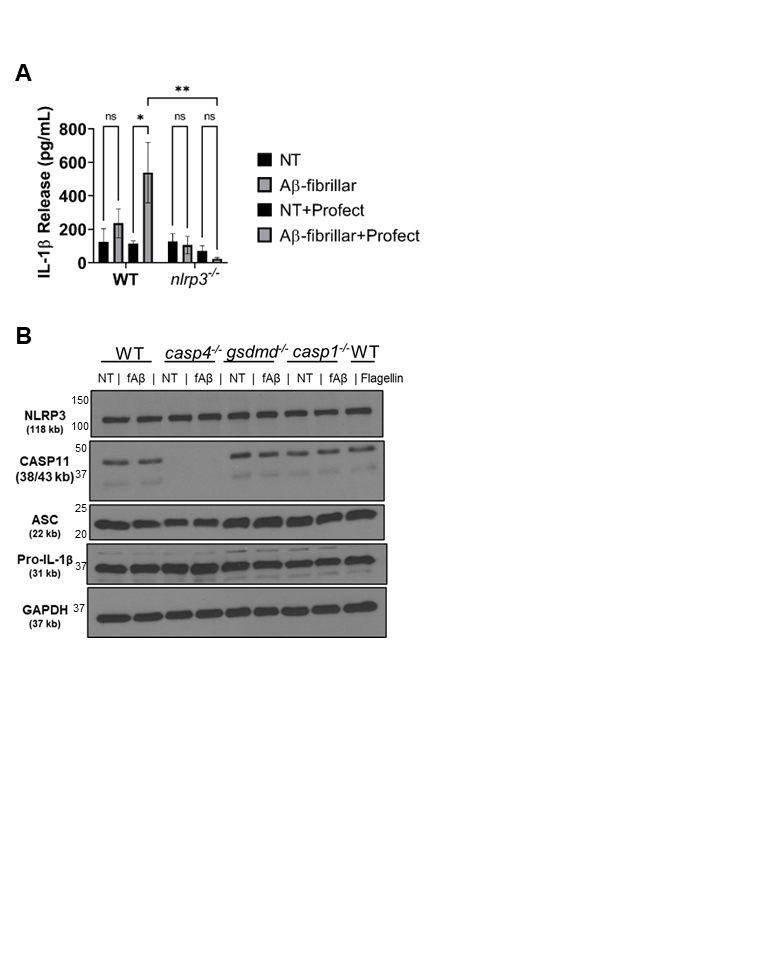

Supplement: Supplementary file 1 — Additional file 1: Supplementary Figure 1. Microglial cells express high levels of Casp4 within mouse brains. A) UMAP visualization of brain cell meta-clusters identified in single-nucleus RNA-sequencing analysis of female mouse brain tissues (GSE207848). UMAP plots showing high RNA expression levels of Casp4 (B) and Cx3cr1 (C) within microglial cells from mouse brain tissues. Supplementary Figure 2. Fibrillar Aβ(1-42) does not prime the inflammasome response or promote cell death. A) IL-1β release from resting macrophages treated for 3 hours with 10µM fibrillar-Aβ (fAβ) or not treated (NT) with and without conjugation to cytosolic delivery reagent Profect or with LPS control followed by 30-minute activation with 5mM ATP (N=4 or N=3 for LPS only). Statistical analysis completed by mixed effects analysis with Tukey’s multiple comparisons test. B) Cell death measured by % LDH release (relative to high control) from LPS-primed mouse macrophages treated for 3 hours with 10µM fibrillar-Aβ with and without Profect, Statistical analysis completed by 2way ANOVA Tukey’s multiple comparisons test (N=5). Supplementary Figure 3. The NLRP3 inflammasome promotes IL-1β release in response to Profect-conjugated-Fibrillar Aβ(1-42). A) IL-1β release from LPS-primed macrophages from wild-type (WT) and nlrp3-/- mice treated for 3 hours with 10µM fibrillar-Aβ or not treated (NT) with and without conjugation to Profect (N=3). B) Representative immunoblots for NLRP3, CASP11, ASC, Pro-IL1β and loading control GAPDH from macrophages lysates treated with Profect-conjugated fibrillar-Aβ (fAβ) or Profect alone (NT) as in Figure 6A (N=2). Statistical analysis completed by 2way ANOVA Tukey’s multiple comparisons test. For simplicity, graph does not display p-values for all comparisons. *P ≤ 0.05, **P ≤ 0.01. Supplementary Figure 4. Expression of inflammasome components in the hippocampus of 5xFAD and 5xFAD/Casp4-/- mice. Immunoblots for CASP1, cleaved CASP1, ASC, Pro-IL1β and GAPDH loading contr [file 13195_2024_1390_MOESM1_ESM.zip › Supplementary Figure 3.tif]

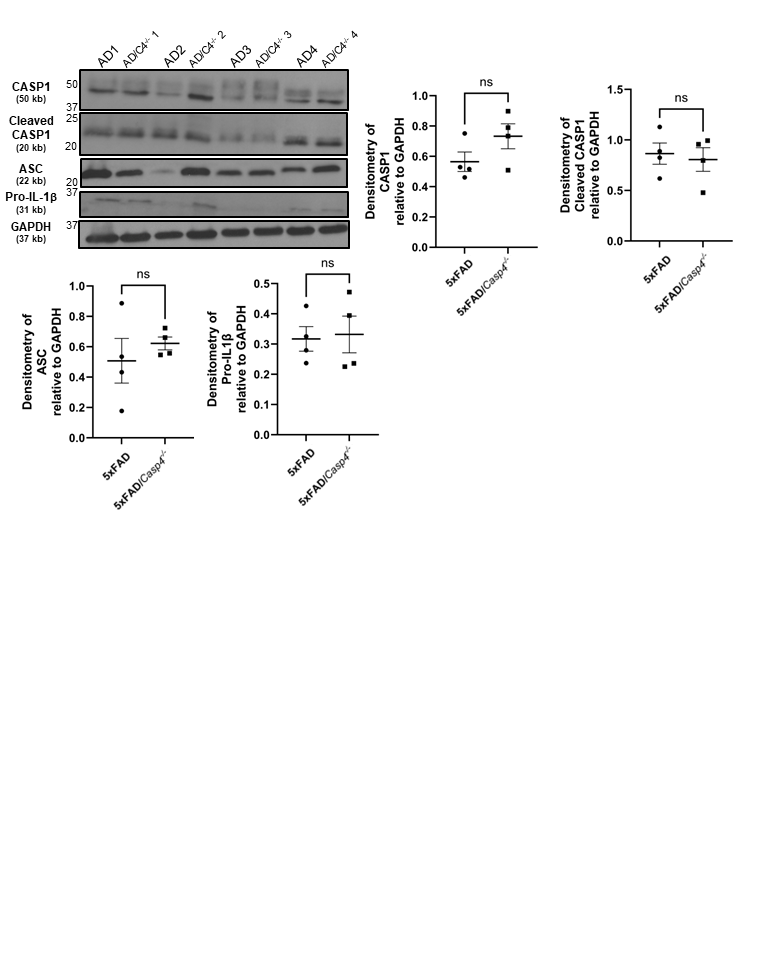

Supplement: Supplementary file 1 — Additional file 1: Supplementary Figure 1. Microglial cells express high levels of Casp4 within mouse brains. A) UMAP visualization of brain cell meta-clusters identified in single-nucleus RNA-sequencing analysis of female mouse brain tissues (GSE207848). UMAP plots showing high RNA expression levels of Casp4 (B) and Cx3cr1 (C) within microglial cells from mouse brain tissues. Supplementary Figure 2. Fibrillar Aβ(1-42) does not prime the inflammasome response or promote cell death. A) IL-1β release from resting macrophages treated for 3 hours with 10µM fibrillar-Aβ (fAβ) or not treated (NT) with and without conjugation to cytosolic delivery reagent Profect or with LPS control followed by 30-minute activation with 5mM ATP (N=4 or N=3 for LPS only). Statistical analysis completed by mixed effects analysis with Tukey’s multiple comparisons test. B) Cell death measured by % LDH release (relative to high control) from LPS-primed mouse macrophages treated for 3 hours with 10µM fibrillar-Aβ with and without Profect, Statistical analysis completed by 2way ANOVA Tukey’s multiple comparisons test (N=5). Supplementary Figure 3. The NLRP3 inflammasome promotes IL-1β release in response to Profect-conjugated-Fibrillar Aβ(1-42). A) IL-1β release from LPS-primed macrophages from wild-type (WT) and nlrp3-/- mice treated for 3 hours with 10µM fibrillar-Aβ or not treated (NT) with and without conjugation to Profect (N=3). B) Representative immunoblots for NLRP3, CASP11, ASC, Pro-IL1β and loading control GAPDH from macrophages lysates treated with Profect-conjugated fibrillar-Aβ (fAβ) or Profect alone (NT) as in Figure 6A (N=2). Statistical analysis completed by 2way ANOVA Tukey’s multiple comparisons test. For simplicity, graph does not display p-values for all comparisons. *P ≤ 0.05, **P ≤ 0.01. Supplementary Figure 4. Expression of inflammasome components in the hippocampus of 5xFAD and 5xFAD/Casp4-/- mice. Immunoblots for CASP1, cleaved CASP1, ASC, Pro-IL1β and GAPDH loading contr [file 13195_2024_1390_MOESM1_ESM.zip › Supplementary Figure 4.tif]

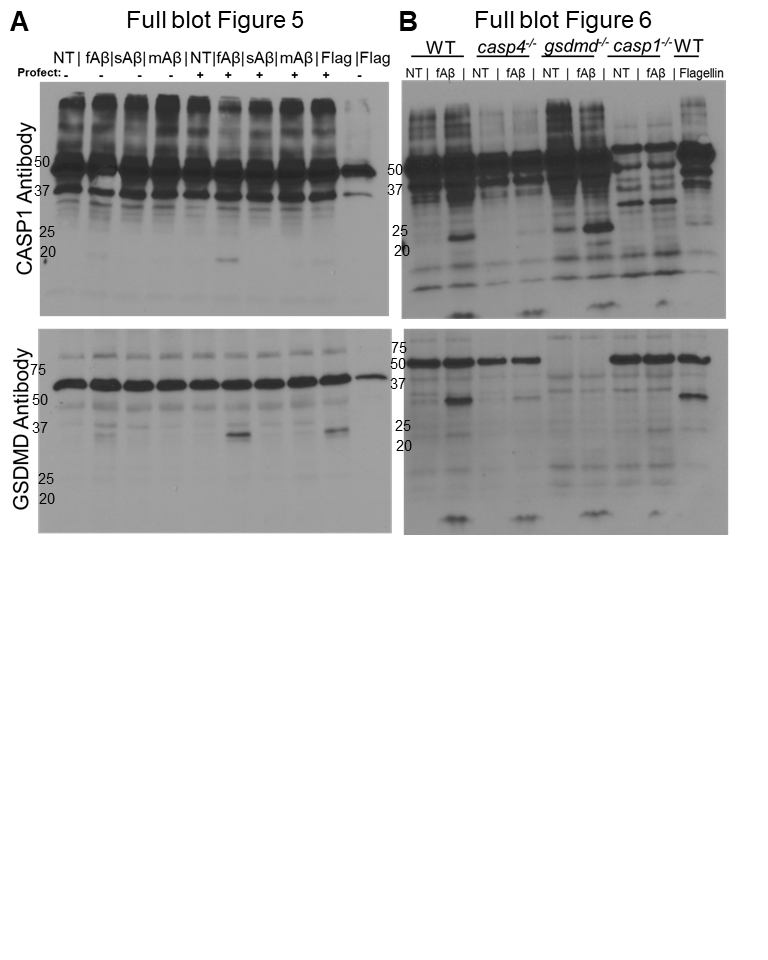

Supplement: Supplementary file 1 — Additional file 1: Supplementary Figure 1. Microglial cells express high levels of Casp4 within mouse brains. A) UMAP visualization of brain cell meta-clusters identified in single-nucleus RNA-sequencing analysis of female mouse brain tissues (GSE207848). UMAP plots showing high RNA expression levels of Casp4 (B) and Cx3cr1 (C) within microglial cells from mouse brain tissues. Supplementary Figure 2. Fibrillar Aβ(1-42) does not prime the inflammasome response or promote cell death. A) IL-1β release from resting macrophages treated for 3 hours with 10µM fibrillar-Aβ (fAβ) or not treated (NT) with and without conjugation to cytosolic delivery reagent Profect or with LPS control followed by 30-minute activation with 5mM ATP (N=4 or N=3 for LPS only). Statistical analysis completed by mixed effects analysis with Tukey’s multiple comparisons test. B) Cell death measured by % LDH release (relative to high control) from LPS-primed mouse macrophages treated for 3 hours with 10µM fibrillar-Aβ with and without Profect, Statistical analysis completed by 2way ANOVA Tukey’s multiple comparisons test (N=5). Supplementary Figure 3. The NLRP3 inflammasome promotes IL-1β release in response to Profect-conjugated-Fibrillar Aβ(1-42). A) IL-1β release from LPS-primed macrophages from wild-type (WT) and nlrp3-/- mice treated for 3 hours with 10µM fibrillar-Aβ or not treated (NT) with and without conjugation to Profect (N=3). B) Representative immunoblots for NLRP3, CASP11, ASC, Pro-IL1β and loading control GAPDH from macrophages lysates treated with Profect-conjugated fibrillar-Aβ (fAβ) or Profect alone (NT) as in Figure 6A (N=2). Statistical analysis completed by 2way ANOVA Tukey’s multiple comparisons test. For simplicity, graph does not display p-values for all comparisons. *P ≤ 0.05, **P ≤ 0.01. Supplementary Figure 4. Expression of inflammasome components in the hippocampus of 5xFAD and 5xFAD/Casp4-/- mice. Immunoblots for CASP1, cleaved CASP1, ASC, Pro-IL1β and GAPDH loading contr [file 13195_2024_1390_MOESM1_ESM.zip › Supplementary Figure 5.tif]
